# Moderate strength (0.23–0.28 T) static magnetic fields (SMF) modulate signaling and differentiation in human embryonic cells

**DOI:** 10.1186/1471-2164-10-356

**Published:** 2009-08-04

**Authors:** Zhiyun Wang, Anshu Sarje, Pao-Lin Che, Kevin J Yarema

**Affiliations:** 1 Department of Biomedical Engineering, The Johns Hopkins University, Baltimore, MD, USA; 2 Department of Electrical and Computer Engineering, University of Maryland, College Park, MD, USA

## Abstract

**Background:**

Compelling evidence exists that magnetic fields modulate living systems. To date, however, rigorous studies have focused on identifying the molecular-level biosensor (e.g., radical ion pairs or membranes) or on the behavior of whole animals leaving a gap in understanding how molecular effects are translated into tissue-wide and organism-level responses. This study begins to bridge this gulf by investigating static magnetic fields (SMF) through global mRNA profiling in human embryonic cells coupled with software analysis to identify the affected signaling pathways.

**Results:**

Software analysis of gene expression in cells exposed to 0.23–0.28 T SMF showed that nine signaling networks responded to SMF; of these, detailed biochemical validation was performed for the network linked to the inflammatory cytokine IL-6. We found the short-term (<24 h) activation of IL-6 involved the coordinate up-regulation of toll-like receptor-4 (TLR4) with complementary changes to NEU3 and ST3GAL5 that reduced ganglioside GM3 in a manner that augmented the activation of TLR4 and IL-6. Loss of GM3 also provided a plausible mechanism for the attenuation of cellular responses to SMF that occurred over longer exposure periods. Finally, SMF-mediated responses were manifest at the cellular level as morphological changes and biochemical markers indicative of pre-oligodendrocyte differentiation.

**Conclusion:**

This study provides a framework describing how magnetic exposure is transduced from a plausible molecular biosensor (lipid membranes) to cell-level responses that include differentiation toward neural lineages. In addition, SMF provided a stimulus that uncovered new relationships – that exist even in the absence of magnetic fields – between gangliosides, the time-dependent regulation of IL-6 signaling by these glycosphingolipids, and the fate of embryonic cells.

## Background

Life exists amid an electromagnetic background and it is therefore not surprising that biological systems are finely tuned to detect and react to static magnetic fields (SMF) of various strengths. In a well known example from nature, the migration of birds is guided by very low strength geomagnetic fields [1-5]. In humans, there are intriguing reports – exemplified by an anecdotal Harvard study that showed severely depressed manic depressive patients experienced dramatic mood swings towards happiness during MRI [6] and pilot pain management clinical trials [7,8] – that magnetic fields can benefit health. In more rigorously controlled animal studies, beneficial effects on pain reduction [9], hypertension [10], wound healing [11], inflammation [12], and microvascular circulation [13] have been reported. To facilitate the translation of these early results to efficacious therapeutic modalities, a greater understanding of the underlying biological basis of magnetic exposure is required [13]. Accordingly, in this paper we take steps towards bridging the gap between the established biophysical effects of magnetic fields on sub-cellular macromolecular components and reported tissue-level and whole organism responses by exploring whether SMF can function as a novel stimulus for signaling pathways at the cell level.

The premise that SMF can modulate signaling networks is based on reports that establish lipid bilayers as the most compelling molecular biosensors capable of responding to magnetic exposure. Specifically, moderate strength SMF can change biophysical properties of membranes that include hyperpolarization [14], redox potential [15], and fluidity [16] thereby altering flux through sodium (Na^+^) [17] and calcium (Ca^2+^) [13,16] channels. As a result, changes in cytosolic concentrations of the calcium ion – which serves as a second messenger in several signaling pathways – occurs ubiquitously in cells exposed to SMF [18]. In addition to altering ion channel flux, biophysical changes to membranes may also affect lipid raft microdomains in ways that modulate downstream signaling; an example of this phenomenon is the impact of ethanol on lipid rafts and the concomitant changes to toll like receptor 4 (TLR4) activity [19]. In contrast to ethanol – which increases membrane domain fluidity – SMF exposure increases membrane rigidity, an effect that has been coupled to the promotion of differentiation in osteoblast-like cells [20].

**Figure 1:**
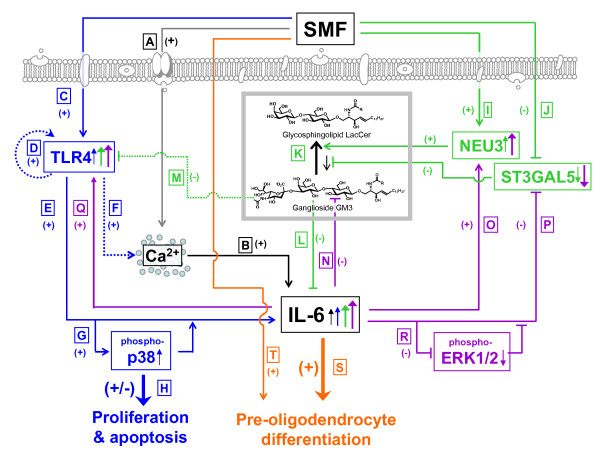
**Overview of crosstalk between SMF and IL-6**. Connections between SMF and IL-6 are shown in black, TLR4 in blue, gangliosides in green, feed-forward regulation of IL-6 on itself in purple, and whole cell responses (e.g., differentiation) in orange. Connections supported by data collected in this work are shown by solid lines (and the location of this data in subsequent figures is provided); dotted lines show connections based on the literature, as referenced. (**A**) SMF exposure modulates calcium flux (Fig. 3C and Fig. 11A). (**B**) Early increases in IL-6 mRNA levels (within 2 h, Fig. 3A) occur followed by increased levels of secreted IL-6 (within 7 h, Fig. 3B). (**C**) Likewise, SMF activates TLR4 (Fig. 3D), resulting in (**D**) feed-forward self-stimulation [41]. (**E**) In turn, TLR4 leads to IL-6 activation either through (**F**) a reported Ca^2+^-dependent route [39] or (**G**) through changes to p38 phosphorylation (Fig. 4A) that (**H**) transiently hinder proliferation in hEBD LVEC cells (Fig. 4B) without leading to apoptosis (Fig. 4C& D). SMF also has early-acting effects on NEU3 (**I**) and ST3GAL5 (**J**) mRNA levels (Fig. 8F) with (**K**) a concomitant decrease in ganglioside levels (Fig. 8B&C). (**L**) In the absence of SMF, exogenously-added ganglioside GM3 suppresses IL-6 production (Fig. 6A) and (**M**) TLR4 [38]. (**N**) IL-6 reduces ganglioside levels (Fig. 6B) through changes to (**O**) NEU3 and (**P**) ST3GAL5 mRNA levels (Fig. 7D) that (**R**) involve ERK1/2 phosphorylation (Fig. 7B&C). 'Downstream' responses to the combined administration of (**S**) IL-6 and (**T**) SMF include reproducible changes in cell morphology (Fig. 9E) and biochemical markers consistent with pre-oligodendrocyte differentiation (Fig. 10).

In the first part this study, mRNA profiling of SMF-treated cells coupled with analysis of the microarray data by the Ingenuity Pathway Analysis software tool [21-23] verified that anticipated transcriptional changes – qualitatively consistent with the impact of altered Ca^2+ ^flux or membrane domain fluidity on signaling pathways – did occur. Building on this finding, we conducted a detailed molecular and biochemical characterization of cellular elements linked to interleukin-6 (IL-6, which was identified to respond to SMF from the software analysis) in human embryonic cells. As a framework for the ensuing experiments described in this study, these connections are diagrammed in Figure 1; this figure shows both known connections between IL-6 and other molecular players (e.g., Ca^2+ ^and TLR4) as well previously unappreciated links (e.g., ganglioside involvement in IL-6 activation that acts even in the absence of SMF, offering a new controlling mechanism for IL-6). This study concludes by showing that SMF leads towards oligodendrocyte differentiation in human embryonic cells by preferentially stimulating pre-oligodendrocyte markers over the astrocyte markers usually associated with IL-6 exposure. Together, these results establish SMF as an intriguing means to ultimately (and non-invasively) stimulate cells in an endogenous niche.

## Results and discussion

### Transcriptional profiling and Ingenuity Pathway Software analysis

To gain evidence for the hypothesis that SMF exposure activates or otherwise modulates signaling networks, human embryoid body derived (hEBD) cells [24] were exposed to 0.23–0.28 T fields and mRNA microarray profiling was used to determine changes to global patterns of gene expression. In the first tests, 15 min SMF exposure (followed by one day recovery) was tested based on reports that gene expression responded to magnetic exposure this quickly [25]. In our evaluation, however, only two genes were up- and down-regulated with a statistical probability > 95% (Table 1) and none met the common benchmark of a 2- (or even 1.75-) fold change. Nonetheless, the reproducibility over multiple probes for the same gene indicated that these modest changes were real and provided impetus to investigate longer term exposure.

**Table 1:** Microarray profiling of mRNA levels in hEBD cells exposed to SMF for 15 min.

Affy Probeset ID	Gene Title	Gene Symbol	Ratio	Average Signals	
			(+ or -) fold-regulation	Control	Experimental
**Up-regulated genes:**					
209189_at	v-fos FBJ murine osteosarcoma viral oncogene homolog	FOS	(+) 1.59	105.4	168.0
1562836_at	DEAD (Asp-Glu-Ala-Asp) box polypeptide 6	DDX6	(+) 1.59	226.5	359.8
205195_at	adaptor-related protein complex 1, sigma 1 subunit	AP1S1	(+) 1.48	212.1	314.4
219730_at	mediator of RNA polymerase II transcription, subunit 18 homolog (yeast)	MED18	(+) 1.47	20.3	29.7
209189_at	v-fos FBJ murine osteosarcoma viral oncogene homolog	FOS	(+) 1.59	105.4	168.0
**Down-regulated genes:**					
AFFX-BioB-3_at	Biotin synthase///biotin synthesis, sulfur insertion	bioB	(-) 1.70	422.5	275.5
AFFX-r2-Ec-bioC-5_at	Biotin synthesis protein bioC///biotin biosynthesis; reaction prior to pimeloyl CoA	bioC	(-) 1.62	1185.5	732.7
AFFX-BioDn-5_at	dethiobiotin synthetase	bioD	(-) 1.52	1181.8	734.2

**Figure 2:**
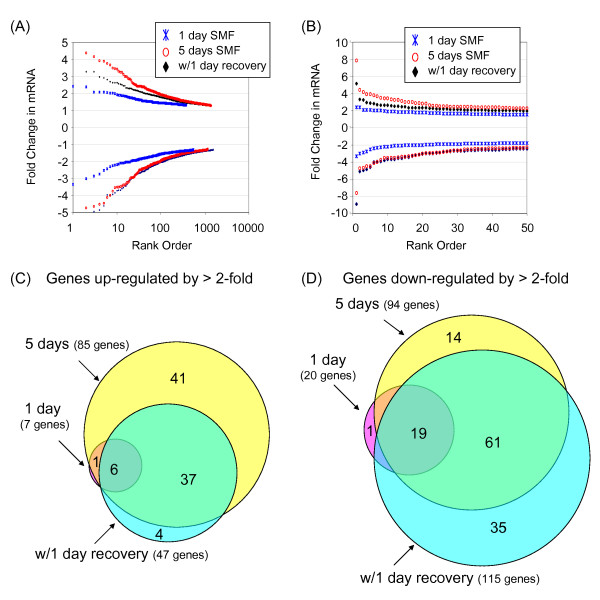
**mRNA profiling of SMF-treated hEBD cells**. (**A**) The magnitude and number of genes that showed statistically significant changes in mRNA levels after SMF exposure compared to untreated control cells are given (four data points, representing genes with ≥ 5-fold changes in mRNA levels are not indicated in (**A**) but are shown in the expanded view of the 50 highest up- and down-regulated genes provided in (**B**); the "missing" genes are listed in Tables 2–4). Venn diagrams depicting the number of genes up- or down-regulated by ≥ 2-fold compared to cells continuously incubated under normal culture conditions are shown in Panels **C **and **D**, respectively. Note that the "1 day SMF" designation refers to the **Group 2 **treatment conditions described in the Methods section, (the greatest up- and down-regulated genes are listed in Table 2); "5 days SMF" refers to **Group 3 **cells (Table 3); and "w/1 day recovery" refers to **Group 4 **cells (Table 4); in all cases comparison is made to **Group 1 **control cells that were not exposed to SMF.

Indeed, after one day (~24 h) of SMF treatment, 379 genes were up-regulated and 549 were down-regulated with statistical significance (Figure 2A); even greater changes were seen after 4 or 5 days of exposure. The magnitude of the change for most genes, however, was modest (Figure 2B) with only 7 showing up-regulation ≥ 2-fold (Figure 2C) and 20 showing a similar degree of down-regulation (Figure 2D). After 5 days of continuous SMF exposure, the number of genes up-regulated by ≥ 2-fold increased to 85 (Figure 2C) while 94 were down-regulated to a similar extent. Interestingly, in an experiment where the cells were allowed to recover for one day under normal culture conditions after prolonged SMF exposure, the number of genes that remained up-regulated by ≥ 2-fold fell by almost half (from 85 to 47, Figure 2C) whereas the number of down-regulated genes increased by 35 (Figure 2D).

The microarray results were consistent with the activation of signal transduction pathways over the short term (i.e., in less than one day) leading to an amplified set of genetic changes over the next several days. A simple inspection of transcriptional changes (for example, the top 5 up- and down-regulated genes under each exposure condition listed in Tables 2, 3, and 4) did not lead to any obvious insights into the over-riding effects of SMF however. Therefore, to flesh out this hypothesis, the Ingenuity Pathway Analysis software tool [21,26] was used to analyze the microarray data resulting in the identification of nine networks that responded to SMF exposure in hEDB LVED cells (Table 5; data analysis is shown for cells subject to five days of continuous SMF exposure and the annotated networks are provided in Additional file 1). Several of these pathways reflected known biological responses to magnetic exposure. For example, changes to intracellular Ca^2+ ^pools observed in cell lines exposed to SMF [18,27] were consistent with interleukin-6 (IL-6) centered signaling responses (ID#2, Table 5) mediated through the ability of this cytokine to be modulated by Ca^2+ ^flux [28]. Similarly, Wnt responses (ID#6, Table 5) can be activated by a non-canonical Ca^2+ ^dependent mechanism [29]. Moving above the cell level, two networks were identified (ID#3 and ID#5) that related to cardiovascular development and hematological function, respectively, and thus dovetail with a recent report by Morris and Skalak where SMF exposure of 0.06–0.14 T for a comparable time period (seven days) facilitated micro-vessel regeneration after surgical intervention [13]. Likewise, Strieth and coauthors have reported that SMF affects the vascular and blood flow [30] and Okano and coworkers have investigated the modulation of blood vessels by magnetic fields [10,31-33].

**Table 2:** Gene expression for hEBD LVEC cells exposed to SMF for one day (i.e., "Group 2") compared to control cells incubated without SMF exposure (Group 1).

Affymetrix Probeset ID	Gene Designation	Gene Symbol	Ratio	Average Signals	
			(+ or -) fold-regulation	Control	Experimental
**Up-regulated genes:**					
1554452_a_at	hypoxia-inducible protein 2	HIG2	(+) 2.41	328.9	792.0
230746_s_at	Stanniocalcin 1	STC1	(+) 2.40	1035.7	2483.9
218149_s_at	zinc finger protein 395	ZNF395	(+) 2.08	349.4	725.9
218507_at	hypoxia-inducible protein 2	HIG2	(+) 2.08	391.7	813.4
223216_x_at	zinc finger protein 395///F-box protein 16	ZNF395///FBXO16	(+) 2.05	241.7	495.4
**Down-regulated genes:**					
217967_s_at	chromosome 1 open reading frame 24	C1orf24	(-) 3.34	848.5	254.0
205569_at	lysosomal-associated membrane protein 3	LAMP3	(-) 3.01	186.3	61.9
229778_at	Hypothetical protein	MGC10946	(-) 2.74	159.6	58.3
209774_x_at	chemokine (C-X-C motif) ligand 2	CXCL2	(-) 2.52	611.4	242.4
220892_s_at	phosphoserine aminotransferase 1	PSAT1	(-) 2.42	2701.91	1116.08
205207_at	interleukin 6 (interferon, beta 2)	IL6	(-) 1.93		266.3

**Table 3:** Gene expression for hEBD LVEC cells exposed to SMF for five days (i.e., Group 3) compared to control cells incubated without SMF exposure (Group 1).

Affymetrix Probeset ID	Gene Designation	Gene Symbol	Ratio	Average Signals	
			(+ or -) fold-regulation	Control	Experimental
**Up-regulated genes:**					
242517_at	G protein-coupled receptor 54	GPR54	(+) 7.84	60.1	470.7
1554452_a_at	hypoxia-i hypoxia-inducible protein 2nducible protein 2	HIG2	(+) 4.40	328.9	1446.4
205493_s_at	dihydropyrimidinase-like 4	DPYSL4	(+) 4.17	184.1	767.3
226682_at	hypothetical protein	LOC283666	(+) 3.91	391.7	1530.5
226431_at	amyotrophic lateral sclerosis 2 (juvenile) chromosome region, candidate 13	ALS2CR13	(+) 3.79	80.9	306.4
232068_s_at	toll-like receptor 4	TLR4	(+) 1.58	195.4	309.5
**Down-regulated genes:**					
217967_s_at	chromosome 1 open reading frame 24	C1orf24	(-) 7.60	848.5	111.7
220892_s_at	phosphoserine aminotransferase 1	PSAT1	(-) 4.72	2701.9	572.5
205047_s_at	asparagine synthetase	ASNS	(-) 4.64	2327.5	501.5
210587_at	inhibin, beta E	INHBE	(-) 4.12	176.6	42.8
204475_at	matrix metallopeptidase 1 (interstitial collagenase)	MMP1	(-) 3.98	112.8	28.3
205207_at	interleukin 6 (interferon, beta 2)	IL-6	(-) 3.23	513.2	158.9

**Table 4:** Gene expression for hEBD LVEC cells exposed to SMF for four days followed by one day of recovery (i.e., Group 4) compared to cells incubated without SMF exposure (Group 1).

Affymetrix Probeset ID	Gene Designation	Gene Symbol	Ratio	Average Signals	
			(+ or -) fold-regulation	Control	Experimental
**Up-regulated genes:**					
242517_at	G protein-coupled receptor 54	GPR54	(+) 5.16	60.1	310.0
205200_at	C-type lectin domain family 3, member B	CLEC3B	(+) 3.28	111.1	364.7
205493_s_at	dihydropyrimidinase-like 4	DPYSL4	(+) 3.27	184.1	601.7
218149_s_at	zinc finger protein 395	ZNF395	(+) 2.96	409.4	1212.0
1554452_a_at	hypoxia-inducible protein 2	HIG2	(+) 2.90	328.9	955.0
232068_s_at	toll-like receptor 4	TLR4	(+) 1.36	195.4	266.5
**Down-regulated genes:**					
217967_s_at	chromosome 1 open reading frame 24	C1orf24	(-) 8.90	848.5	95.3
205047_s_at	asparagine synthetase	ASNS	(-) 4.95	2327.5	470.0
223062_s_at	phosphoserine aminotransferase 1	PSAT1	(-) 4.84	3784.5	781.5
210587_at	inhibin, beta E	INHBE	(-) 3.96	176.6	44.6
229778_at	Hypothetical protein	MGC10946	(-) 3.95	159.6	40.5
205207_at	interleukin 6 (interferon, beta 2)	IL6	(-) 3.16	513.2	162.4

**Table 5:** Signaling networks identified to respond to SMF exposure through data analysis with the Ingenuity Pathway Analysis software tool.^1^

ID #	Signaling Network Title
1	Lipid Metabolism, Small Molecule Biochemistry, Drug Metabolism
2	Metabolic Disease, Cellular Development, Connective Tissue Development and Function
3	Cardiovascular System Development and Function, Cellular Development, Cellular Growth and Proliferation
4	Cell Cycle, Organ Morphology, Connective Tissue Development and Function
5	Cellular Movement, Cell-To-Cell Signaling and Interaction, Hematological System Development and Function
6	Cell Death, Neurological Disease, Cellular Function and Maintenance
7	Lipid Metabolism, Small Molecule Biochemistry, Metabolic Disease
8	Carbohydrate Metabolism, Molecular Transport, Small Molecule Biochemistry
9	Cell Cycle, Cancer, Cellular Growth and Proliferation

^1 ^Annotated network diagrams are provided in Additional file 1.

### SMF increased IL-6 mRNA levels and protein secretion at early time points

Even though the software analysis of the microarray data was consistent with a mechanism wherein SMF acted as a stimulus for signaling pathways, limitations of this methodology precluded any firm conclusions. Signaling pathway responses, for example, are typically measured over time intervals of minutes to hours and require evaluation with closely-spaced time points not practical by microarray profiling over several days. Therefore, to verify that the transcriptional changes we observed represented legitimate responses to SMF, we selected IL-6 for conventional biochemical characterization. Of the nine networks identified by microarray profiling, the selection of IL-6 for additional scrutiny was based on several factors. First, a recent report linked 0.4 T SMF exposure to increased IL-6 production in fibroblasts [34] and plausible membrane-based modes of activation IL-6 (e.g., through Ca^2+ ^or TLR4) exist. Furthermore, reports that SMF can promote differentiation [20] – coupled with the propensity of the hEBD LVEC line used in this study to display neural markers [24] together with reports that IL-6 promotes astrocytogenesis [35] – offered the possibility that cell-level responses (e.g., differentiation of the hEBD cells to astrocytes) could be observed in these experiments.

**Figure 3:**
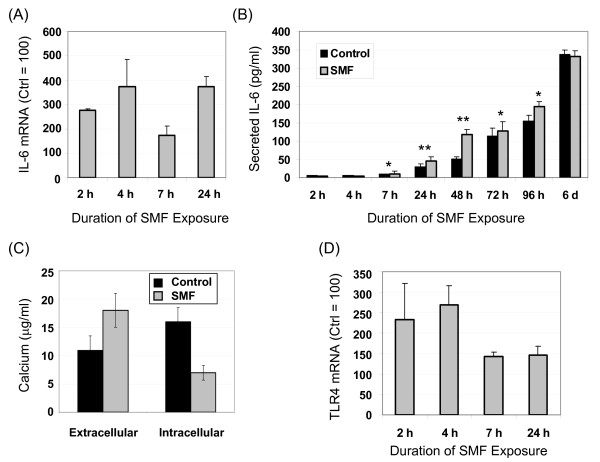
**Response of interleukin-6 (IL-6) to SMF exposure and putative activating mechanisms in hEBD LVEC cells**. (**A**) Levels of IL-6 mRNA were determined by qRT-PCR during the first 24 h of SMF exposure. (**B**) IL-6 concentrations in the culture medium of SMF-exposed and control cells were determined by ELISA at the indicated times. (**C**) Levels of extracellular and intracellular calcium after four hours of SMF exposure show the expected reciprocal relationship (a time course for extracellular calcium is shown in Figure 11A). (**D**) Levels of TLR4 mRNA were determined by qRT-PCR during the first 24 h of SMF exposure. In all cases the data shown represents three or more independent experiments (denoted as *n *≥ 3 in subsequent figure legends) and error bars represent standard (SD); in Panels **A**, **C**, and **D**, *p *< 0.05 for all data points shown that compare SMF-treated and control cells; in Panel **B**, "*" indicates *p *< 0.05 and "**" indicates *p *< 0.01 (in this figure, and throughout this study, statistical significance was determined by the Student t-test and differences were considered to be significant when *p *< 0.05 (or *p *< 0.01 when specifically indicated)).

Biochemical validation began by quantitative real-time polymerase chain reaction (qRT-PCR) analysis of IL-6 mRNA levels over the first 24 h of SMF exposure, a time frame selected based on the numerous changes seen in the microarray data after one day (Figure 2) and literature reports of biphasic IL-6 activation during this time period [36]. In these experiments, IL-6 mRNA levels increased two hours into SMF exposure and remained elevated compared to untreated control cells at 4, 7 and 24 h (Figure 3A). IL-6 secretion into the culture medium followed slower kinetics, first showing a measurable increase at 7 h after which SMF-treated cells out-produced control cells up to 96 h (Figure 3B). The SMF-exposed cells experienced the largest relative increase compared to untreated controls at 48 h, followed by a decline to slightly less than control levels at the end of the six day monitoring period.

### TLR4 was activated by SMF in tandem with IL-6

Upon verifying that IL-6 was activated by SMF at both the mRNA and protein levels, we sought more detailed insight into this response. As indicated in Figure 1A &1B (for perspective, Figure 1 summarizes the connections between SMF, IL-6 and other pathways elements and cellular outcomes described in this report), IL-6 activation was consistent with the known ability of magnetic fields to alter calcium ion channel flux and reports of Ca^2+^-dependent up-regulation of IL-6 (the impact of SMF on calcium flux was experimentally verified for currently-used hEBD LVEC cells, Figure 3C). In addition, connections IL-6 shares with TLR4 [37-39], combined with the dependence of the signaling activity of Toll-like receptors on their lateral diffusion within membrane microdomains [19,40], suggested a parallel route through which SMF could influence IL-6. Specifically, a sequence of events can be postulated where SMF changes membrane fluidity thereby modulating TLR4 (Figure 1C) and downstream IL-6 responses (Figure 1E) through a Ca^2+^-dependent mechanism (Figure 1F) or through TLR4-mediated p38 phosphorylation (Figure 1G).

Experimentally, because TLR4 transcription is strictly auto-regulated in a stimulus-dependent manner ([38,41] and Figure 1D), qRT-PCR can be used to monitor its activation. By monitoring this endpoint, we found that TLR4 transcript levels increased during the first several hours of SMF exposure (Figure 3D). Interestingly, self-activation of TLR4 can lead to either the down-regulation of its mRNA (as seen in rat glial [38] or murine macrophages [42]) or to up-regulation (as seen in murine lung [37] or human monocytes and polymorphonuclear leukocytes [43]); the current up-regulation of TLR4 mRNA observed in SMF-treated embryonic cells is consistent with results obtained in other types of human cells upon activation of TLRs.

### SMF activation of TLR4 impinges upon MAPK pathways

**Figure 4:**
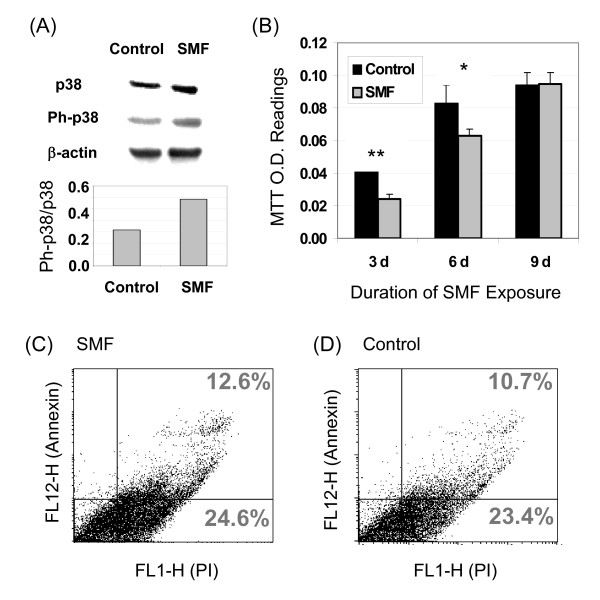
**MAPK-related responses to SMF in hEBD LVEC cells**. (**A**) An increase in p38 phosphorylation occurred in the SMF-treated cells (representative results from one of three experiments is shown for 30 min of SMF exposure; a normalized ratio of phosphorylated p38 (Ph-p38) to p38 is provided in the bar graph after quantification of the western blots by densitometry). (**B**) Proliferation of SMF-exposed cells decreased over the first 6 days of SMF exposure but not at nine days (error bars represent SD for *n *≥ 3 independent experiments and "*" indicates *p *< 0.05 and "**" indicates *p *< 0.01). No change in the number of apoptotic cells was detected after SMF exposure when evaluated by the Annexin V/propidium iodide assay (Panel **C**; representative data from one of three experiments is shown) compared to control cells (Panel **D**).

The activation of IL-6 in cells exposed to SMF was consistent with signal transduction through the upstream involvement of TLR4 (Figure 1E&1G). To gain biochemical evidence for this connection, we analyzed the phosphorylation of p38, which lies in the pathway that connects TLR4 with IL-6, and found the predicted increase in phosphorylated p38 in SMF treated cells (Figure 4A). This result, in addition to establishing a connection between IL-6 and SMF through TLR4, provided evidence that SMF impinges on MAPK signaling (p38 plays a central role in mediating MAPK responses) prompting us to evaluate changes to proliferation and apoptosis. In these experiments a significant reduction in proliferation was seen for hEBD LVEC cells after three days of SMF exposure; this effect lessened by the sixth day and was lost by the ninth day (Figure 4B). Qualitatively, this short term change in proliferation was consistent with studies where SMF transiently altered proliferation [44]. Annexin/propidium iodide staining assays showed that reduced proliferation during early phases of SMF exposure was not a consequence of increased apoptosis (Figure 4C&4D) in agreement with reports that SMF, if anything, is protective against apoptosis [18,45]. Having ruled out that the SMF treated cells were dying, a plausible explanation for the reduced proliferation was that the cells were undergoing differentiation with a concomitant decrease in their growth rate; this possibility was supported by data presented later in this report.

### SMF responses are cell line dependent

**Figure 5:**
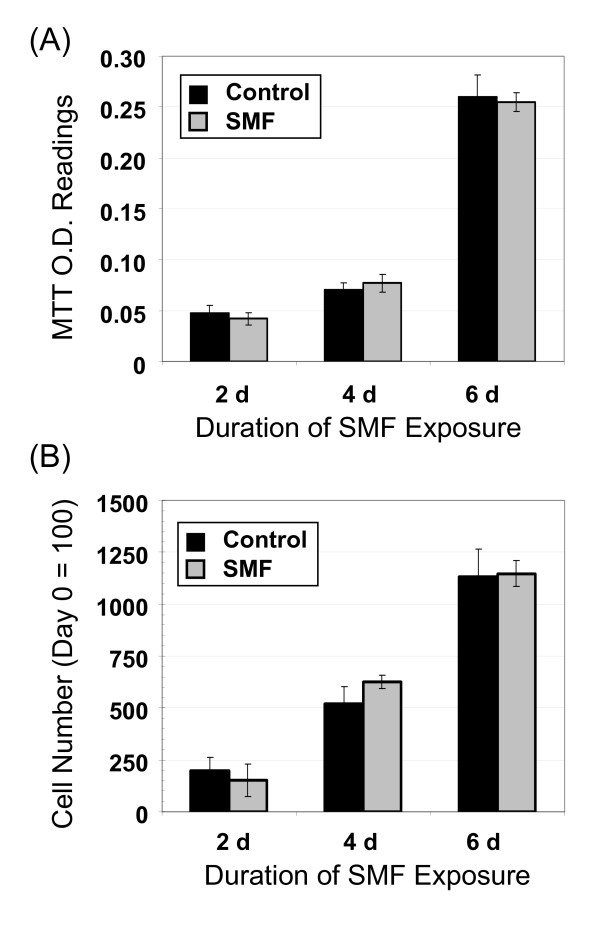
**Proliferation is not altered by SMF in HEK AD293 cells**. The cell line dependence of SMF effects is illustrated by the lack of a response in SMF-treated HEK AD293 cells when proliferation was measured by the MTT assay (Panel **A**) or by cell counts (Panel **B**). Error bars represent SD for *n *≥ 3 independent experiments and in all cases *p *> 0.05.

As a brief diversion from the main thrust of this study, which was to connect SMF with cellular responses associated with IL-6 in human embryonic cells, we wish to emphasize that the impact of SMF on other common laboratory cells such as the Jurkat, HeLa, and HEK AD293 lines was surveyed and "obvious" effects such as pronounced changes in proliferation (as seen in Figure 4B for the hEBD LVEC line) or altered morphology (as shown later in this report) were not observed. For example, representative data is shown for the HEK AD293 line in Figure 5 where control and SMF-exposed cells had identical growth rates when measured by either the MTT assay (Panel A) or through cell counting (Panel B). The clear-cut differences seen between the embryonic hEBD LVEC line and cancer lines were not surprising based on reports that even closely-matched cell lines respond uniquely to SMF [46]; instead these findings support the hypothesis that changes to Ca^2+ ^flux (as shown in Figure 3C) – a parameter that is highly cell line dependent [18] – contributes to the cellular responses we observed in the cells exposed to SMF.

### Connections between gangliosides and IL-6 exist in hEBD LVEC cells

To gain insight into whether regulatory networks beyond TLR4 or calcium flux contributed to the up-regulation of IL-6 in SMF treated cells, we next focused on gangliosides [38,47]. Gangliosides are sialic acid-bearing glycosphingolipids (GSLs) that are integral components of lipid rafts and caveolae of the type surrounding TLR4 that not only organize these microdomains but also regulate the signaling functions of embedded proteins (as discussed in more detail in review articles [48,49]). Consequently, TLR4 [38] and IL-6 [47] can be influenced by the equilibrium between the 'inert' (in this context) GSL lactosylceramide (LacCer) and the suppressive ganglioside GM3 (Figure 1K).

**Figure 6:**
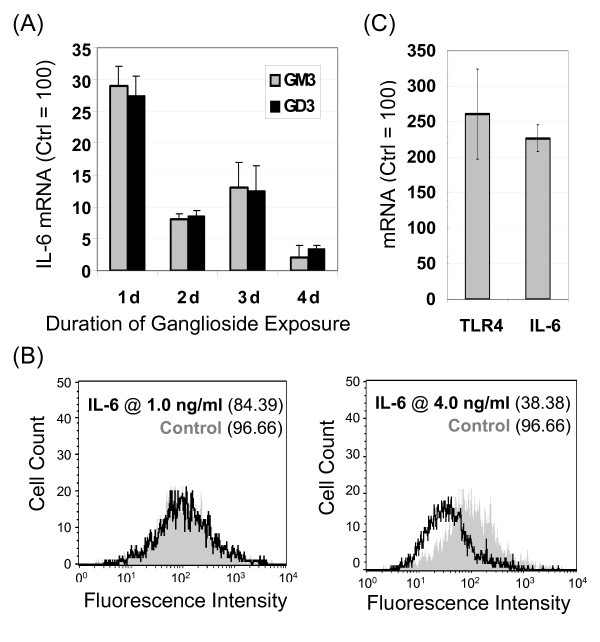
**Crosstalk between IL-6 and gangliosides in hEBD LVEC cells**. (**A**) IL-6 mRNA decreased upon supplementation of the culture medium with 5.0 μM of either GM3 or GD3 (*n *≥ 3; *p *< 0.01 for all time points shown compared with controls that were incubated in the absence of IL-6). (**B**) Ganglioside GM3 decreased in a dose-dependent manner in cells supplemented with 1.0 or 4.0 ng/ml IL-6 for 24 h. (**C**) mRNA levels for IL-6 and TLR-4 increased in cells incubated with IL-6 (error bars represent SD for *n *≥ 3 independent experiments and *p *< 0.05 for both measurements).

Before beginning experiments to probe the impact of SMF exposure on gangliosides, the relationship between GM3 (and its disialylated derivative GD3) and IL-6 was first investigated to establish a baseline for the hEBD LVEC line (before the current study, there was negligible literature precedent for a connection between GSL and IL-6 in human embryonic cells). A long-lasting and substantial (e.g., > 95% at 4 d) reduction in IL-6 mRNA was observed in cells incubated with exogenously-added GM3 or GD3 (Figure 6A). Crosstalk between gangliosides and IL-6 also held in the reverse direction as demonstrated by a dose dependent decrease in GM3 in cells incubated with exogenously-added IL-6 (Figure 6B). By reducing the amount of GM3 present in a cell (Figure 1N), IL-6 can alleviate the suppressive effects of this ganglioside on its transcription (Figure 1L) thus setting up a 'feed-forward' loop that offers an mechanistic explanation for the self-activation of IL-6 described in the literature [50] and demonstrated for hEBD LVEC cells in this study (Figure 6C). Figure 6C shows that levels of TLR4 mRNA also increased significantly in IL-6 supplemented cells consistent with the removal of concomitant inhibitory effects of GM3 on TLR4 [38]. Together with the impact of SMF on IL-6 shown in Figure 3, these results demonstrate that SMF has the capacity for tuning IL-6 signaling by adjusting the relative proportions of the 'active' ganglioside GM3 and its 'inert' asialo counterpart LacCer (Figure 1K) thereby contributing to the transcriptional up-regulation of TLR4 and IL-6 (Figure 1M&1N).

### IL-6 mediated changes to GM3 and GD3 occur via NEU3 and STGAL5

**Figure 7:**
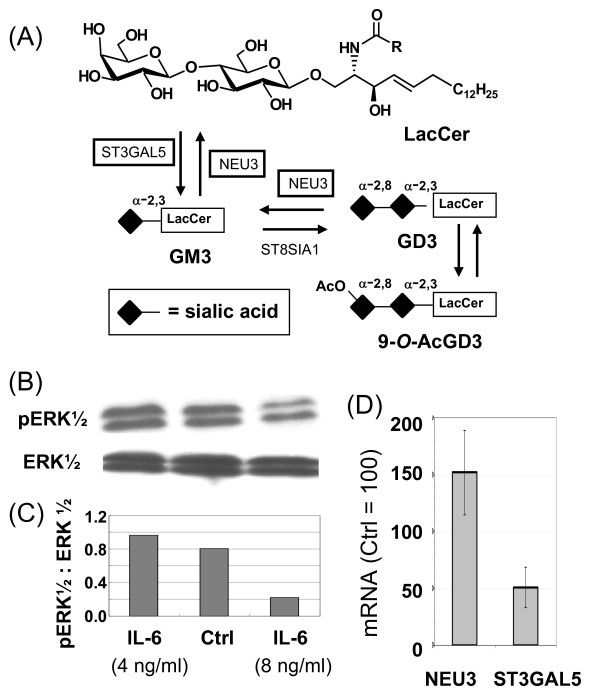
**Ganglioside metabolism and connections with ERK1/2**. (**A**) This diagram shows the conversion of LacCer to the ganglioside GM3 by ST3GAL5 (and then to GD3 upon α-2,8-sialylation and to 9-*O*-AcGD3 upon *O*-acetylation); both GM3 and GD3 are converted back to LacCer by NEU3. (**B**) Western blots of ERK1/2 and pERK1/2 from control and IL-6-treated hEBD LVEC cells (for 30 min) show reduced ERK1/2 phosphorylation at concentrations > 4 ng/ml (as quantified by densitometry and shown as a ratio of pERK1/2 to ERK in Panel **C**). (**D**) IL-6 treated cells exhibited increased levels of NEU3 mRNA and decreased levels of ST3GAL5 mRNA after 24 h (error bars represent SD for *n *≥ 3 independent experiments and *p *< 0.05 for both data sets).

Mechanistically, changes to one of two enzymes could explain the shift in equilibrium away from the suppressive ganglioside GM3 to its inert asialo counterpart LacCer (Figure 1K); specifically, an increase in the recycling enzyme NEU3 or a decrease in the biosynthetic enzyme ST3GAL5 (Figure 7A). Despite no previously-known direct links between IL-6 and ST3GAL5 or NEU3, increased phosphorylation of ERK1/2 has been connected with the up-regulation of STGAL5 ([51], as shown in Figure 1P). Therefore, based on a report linking IL-6 and MAPK signaling through JAK/STAT that involved ERK1/2 (Figure 1R) [52], we reasoned that ERK1/2 could serve as an intermediary to connect IL-6 with ST3GAL5 expression. Accordingly, we tested the phosphorylation of ERK (Figure 7B) and found that pERK1/2 was inhibited by concentrations of IL-6 > 4.0 ng/ml (Figure 7C); the reduced ratio of pERK1/2 to ERK was consistent with dampened mRNA levels for the biosynthetic enzyme ST3GAL5 and the tandem up-regulation of the recycling enzyme NEU3 (Figure 7D). A noteworthy aspect of this study was that, although the effects of NEU3 and ST3GAL5 on "lubricating signaling pathways" [49] have been previously evaluated separately, to our knowledge this is the first report where both enzymes were monitored simultaneously and found to respond to an external stimulus in a concerted manner that required transcriptional regulation of the biosynthetic and recycling enzymes in opposite directions.

The prolonged down-regulation of ganglioside GM3 upon IL-6 supplementation (Figure 6A) provides a two-pronged mechanistic explanation for long term attenuation of IL-6 and related responses in SMF-treated cells (for example, SMF-enhanced IL-6 levels returned to normal by day 6 (Figure 3B) followed by loss of growth inhibition by day 9 (Figure 4B)). First, the loss of sialic acid – an important contributor to the carbohydrate-carbohydrate binding interactions that stabilize lipid assemblies [48] – from GM3 can destabilize CD82-enriched microdomains [53]. Assuming that the TLR4 receptor complex, which is also sensitive to the stability of its local microdomain environment [19], responds to a reduction in GM3 levels in a similar manner, the signaling pathways activated by SMF over the first day or so of exposure could be 'turned off' by the loss of GM3 over longer time periods. A second mechanism to explain ganglioside-mediated attenuation of IL-6 can be postulated based on the findings by Müthing and colleagues that GSL such as GM3 increase Ca^2+ ^flux through voltage gated channels [54]. In an independent set of experiments, Yang and coworkers reported a strongly stimulatory effect for GM3 on the SR Ca^2+-^ATPase [55-58]. Together, these findings indicate that the conversion of GM3 to LacCer in SMF-treated cells inhibits Ca^2+^-dependent signaling pathways in a manner that attenuates the initial multi-pronged up-regulation of IL-6.

### SMF regulates ganglioside production via NEU3 and ST3GAL5

**Figure 8:**
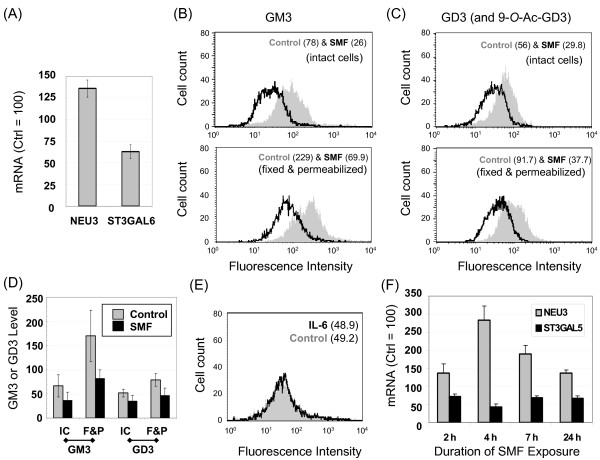
**Effects of SMF on gangliosides GM3 and GD3 and IL-6**. (**A**) mRNA levels increased for NEU3 and decreased for ST3GAL3 during the first 24 hours of SMF exposure consistent with a flow cytometry-measured reduction in GM3 (Panel **B**) and GD3/9-*O*-AcGD3 levels (the antibody used recognized both the 9-*O*-acetylated and hydroxyl forms of GD3, Panel **C**). (**D**) Data from three independent flow cytometry experiments are summarized; GM3 or GD3 levels are given as measured in the arbitrary units shown on the x-axis of Panels **B **and **C**. (**E**) IL-6, which led to a decrease in ganglioside levels in naïve cells (*see *Figure 6A) had no further effect on GM3 levels (or GD3 levels, not shown) in SMF-treated cells. (**F**) A time course of NEU3 and ST3GAL5 shows up- or down-regulation, respectively, over time points shorter than 24 h. Error bars for Panels **A**, **D**, and **F **represent SD for *n *≥ 3 independent experiments and *p *< 0.05 for all data points that compare SMF-treated samples with the matched untreated controls.

The crosstalk between gangliosides and IL-6 (as summarized in Figures 1L&1N and 7A), combined with the ability of SMF to modulate this cytokine (as shown by the data in Figure 3), led us to consider whether SMF altered IL-6 via a ganglioside-mediated route (or vice versa). To investigate this possibility, NEU3 and ST3GAL5 – the enzymes that control the equilibrium between GM3 and LacCer (Figure 7A) and thus have the potential to indirectly modulate IL-6 (Figure 1L&1N) – were monitored by qRT-PCR during the early stages of SMF exposure. In these experiments, up-regulation of NEU3 and inhibition of ST3GAL5 after one day of SMF exposure (Figure 8A) reminiscent of the effects of IL-6 supplementation (Figure 7D) were observed. Analysis of ganglioside levels in these cells showed that these transcriptional changes again worked in concert to decrease GM3 levels on the cell surface (Figure 8B, top). A similar reduction in GM3 occurred in fixed and permeabilized cells where gangliosides situated in the secretory pathway are also measured (Figure 8B, bottom). By testing both conditions, the possibility that surface changes merely reflected the redistribution of GM3 between the cell surface and intracellular compartments was discounted (this concern was raised by the hypothesis that SMF changes the biophysical properties of lipid bilayers thereby potentially affecting trafficking between surface and intracellular membranes). Interestingly, GD3 – which can modulate the biophysical properties of membrane raft assemblies similar to GM3 (and in essence serves as a reservoir for this monosialylated ganglioside, Figure 7A) – was also reduced by SMF (Figure 8C); this result can be explained by the ability of NEU3 to remove both sialic acid residues of GD3.

### SMF regulates NEU3 and ST3GAL5 independently of IL-6

In order to gain insight into the cause and effect relationships that connect SMF, gangliosides, and IL-6, IL-6 was added to cells in the presence or absence of SMF. In this experiment IL-6 had the same effect on GM3 levels with or without concomitant magnetic exposure (Figure 8E). This result contrasted with the clear reduction in GM3 when IL-6 had been added to cells in the absence of SMF (as shown in Figure 6A). One explanation for these disparate results was that SMF activated a sequence of events where IL-6 transcription was first up-regulated leading to increased protein secretion, which in turn reduced GM3. This scenario, however, was discounted by a time course of NEU3 and ST3GAL5 mRNA expression over the first day of SMF exposure (Figure 8F) that showed that the transcriptional changes to these enzymes occurred *before *measurable IL-6 secretion took place (e.g., before 7 h, see Figure 3B). Therefore, SMF *independently *regulates IL-6 and gangliosides in a way that ultimately impinges on the same molecular mechanism (i.e., through NEU3 and ST3GAL5 transcription and activity). GM3 and GD3 also provide a putative explanation for the biphasic increase in IL-6 mRNA; at early time points a ganglioside-independent sequence of events (presumably involving, but not necessarily limited to, TLR4 activation or Ca^2+ ^flux) occurs. As the initial signal fades, reduction of GM3 and GD3 could contribute to a second 'burst' of IL-6 expression by alleviating the suppressive effects of these gangliosides on IL-6 itself (Figure 1L) or on TLR4 (Figure 1M).

### SMF – in combination with IL-6 – alters cell morphology

**Figure 9:**
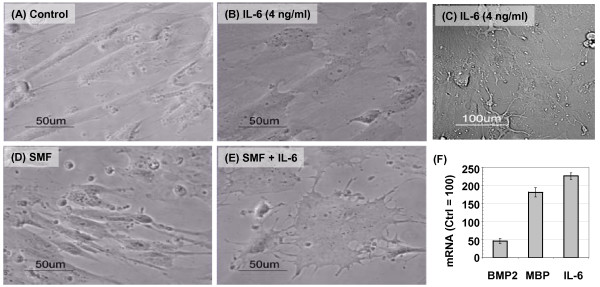
**Morphological effects of SMF and IL-6 on hEBD LVEC cells**. (**A**) Phase contrast images showing the normal morphology of ~90% confluent hEBD LVEC cells. (**B**) Slightly altered morphology typically was seen after incubating the cells with 4.0 ng/ml of IL-6 for 3 days. (**C**) Cells incubated with 4.0 ng/ml IL-6 sporadically attained dramatically altered cell morphology, with neurite-like outgrowths. (**D**) SMF, by itself, had a minor impact on the morphology of hEBD LVEC cells. (**E**) The combination of SMF and 4.0 ng/ml IL-6 resulted in ~100% of the cells attaining the distinctively altered morphology shown. (**F**) mRNA levels of bone morphogenetic protein 2 (BMP2), myelin basic protein (MBP), and IL-6 after 24 h of SMF exposure (error bars represent SD for *n *≥ 3 independent experiments and *p *< 0.05 for each marker).

As described earlier, hEBD LVEC cells exposed to SMF experienced reduced proliferation without toxicity (Figure 4), a response consistent with differentiation. To test if this phenomenon was linked to SMF or IL-6 production, cells were first treated with ≤ 4.0 ng/ml of IL-6 in the absence of SMF. IL-6 supplementation typically resulted in relatively minor (if any) change to cell morphology (Figure 9A&9B). Occasionally, however, dendrite-like outgrowths reminiscent of neuronal cells developed in sub-populations of IL-6 treated cells (Panel C). By contrast, close to 100% of the cells attained distinctive morphology when SMF was combined with 4.0 ng/ml of IL-6 (Panel E; SMF alone had a much less pronounced impact on morphology, Panel D).

One explanation for why both SMF and exogenous IL-6 supplementation was needed to elicit noticeable changes to cell morphology was that, because of the relatively small volume of cells (≤ 0.01%) compared to culture medium, any IL-6 secreted in response to SMF would be diluted ~10,000-fold. As a consequence, additional IL-6 supplementation was required to mimic levels achieved by comparable rates of IL-6 production in cells situated within an *in vivo *niche where the relative cell to interstitial volume ratios are much lower. Another (non-exclusive) explanation, supported by experiments where even 20 ng/ml IL-6 could not reproduce the combined effects of SMF plus 4 ng/ml IL-6 (data not shown), was that SMF-activated networks beyond IL-6 – such as those listed in Table 5 – contributed to the morphological changes.

### SMF promotes oligodendrocyte progenitor markers

**Figure 10:**
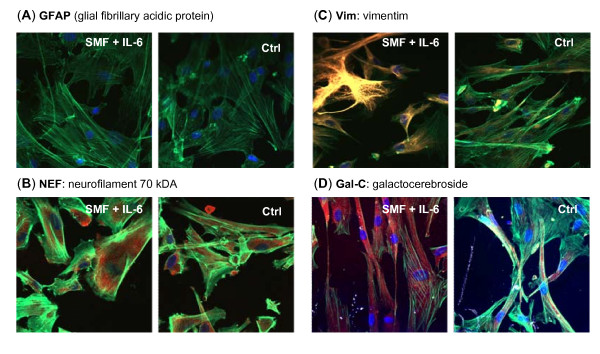
**Differentiation markers in SMF- and IL-6-treated hEBD LVEC cells**. Test cells were incubated with 4.0 ng/ml IL-6 with concurrent SMF exposure (controls were incubated with neither) and the monolayers were co-stained with Oregon Green 488 phalloidin to visualize actin, the nuclear dye DAPI (blue), and one of the following markers (red): (**A**) the astrocyte marker GFAP (note that the lack of staining indicates the absence of this marker in both treated and control cells); (**B**) the neuron marker NEF; and the pre-oligodendrocyte markers (**C**) Vim and (**D**) Gal-C. Images were obtained by confocal microscopy using identical exposure settings for each set of photographs.

To gain greater insight into the morphological changes induced in hEBD LVED cells by a combination of SMF and IL-6, we noted that IL-6 has a role in the regeneration of nervous tissue, usually promoting astrocyte formation [35] and, accordingly, monitored the transcription of bone morphogenic protein 2 (BMP-2) and myelin basic protein (MBP) (Figure 9F). Interestingly, a decrease in mRNA for BMP-2, a protein that stimulates astrocytogenesis [59], was observed suggesting that the hEBD cells were not differentiating into astrocytes as expected. To confirm this observation using immunofluorescent microscopy, no increase in the GFAP marker associated with astrocyte formation was observed in SMF and IL-6 treated cells (Figure 10A). Similarly, no increase was seen for NEF (Figure 10B), a marker associated with neuron differentiation.

Based on the lack of astrocyte or neuron differentiation, a third possibility was that the decrease in BMP-2 expression in SMF-treated cells removed the obstacle presented by bone morphogenetic proteins towards differentiation to oligodendrocyte lineages [60]. Indeed, consistent with the decrease in BMP-2, an increase in myelin basic protein (MBP) transcription was observed (Figure 9F) providing a biochemical marker consistent with differentiation to an oligodendrocytes [61]. Additional supporting evidence that SMF, combined with IL-6, leads toward oligodendrocyte progenitor formation was provided by the increased expression of vimentim (Figure 10C) and Gal-C (Figure 10D).

### A timeline of SMF responses: Towards unraveling cause and effect relationships

**Figure 11:**
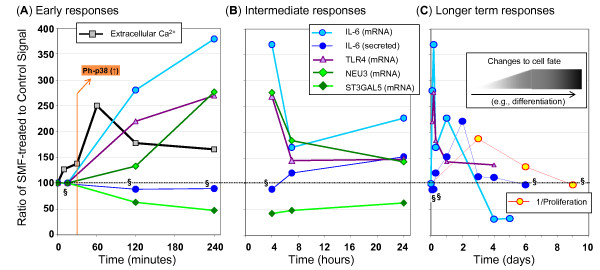
**Timeline of SMF-induced, IL-6 associated responses in hEBD LVEC cells**. Data is compiled from experiments reported throughout this paper to show (**A**) early responses that occur within four hours of the start of continuous SMF exposure, (**B**) intermediate responses that occur over the first day, and (**C**) longer term responses over the first week or so of SMF exposure. Data is shown for *n *≥ 3 independent experiments and *p *< 0.05 for all data except for that indicated by "§" where *p *> 0.05 (these data were analyzed by SD but error bars are omitted from these graphs for clarity). All data shown – except for the proliferation data in Panel **C **that gives the reciprocal relationship – compares SMF-exposed to control cells with a value of 100 as a baseline.

The relationships between SMF, calcium, TLR4, gangliosides (and regulatory enzymes), MAPK pathway elements (p38 and ERK1/2) and IL-6 are outlined in Figure 1; this diagram, however, does not provide a dynamic view that would provide insight into cause and effect relationships. Therefore, to summarize the time dependence of various aspects of hEBD LVEC cell responses to SMF exposure, early, intermediate, and longer term responses are summarized in Figure 11. During the first four hours (Panel A), changes to calcium flux occur within minutes as do MAPK responses (e.g., p38 phosphorylation, Figure 4A); effects on the transcription of IL-6, TLR4, NEU3, and ST3GAL5 mRNA lag slightly but show a strong response beginning between 2 and 4 hours. By contrast, secreted IL-6 remains unchanged. During the remainder of the first day of exposure (Panel B), mRNA levels of SMF treated cells trend back to control levels (the one exception is the 24 hour point for IL-6, which rebounds after a decline between 4 and 7 hours, this biphasic response mimics the impact of other stimuli on IL-6 [36]). Also during the first day, while the impact of SMF on transcription of IL-6, TLR4, NEU3, and ST3GAL5 abates, phenotypic effects such as the accumulation of measurable levels of secreted IL-6 began to be manifest. In general, initiating events – for example, the impact of SMF on mRNA levels – were attenuated after the second day (as shown in Panel C) whereas "behavioral" responses (such as the secretion of IL-6 or the effects of SMF on proliferation) followed the same trend but lagged in time. During this multiday time period – while "intermediate" responses were returning to normal – long-lived changes to cell fate arose that included the morphological changes shown in Figure 9 and the accumulation of pre-oligodendrocyte markers shown in Figure 10.

Another lesson learned from the data shown in Figure 11 was that SMF treatment set in motion a complex sequence of events that rapidly changed over time; consequently, the original goal of analyzing cellular responses by microarray profiling could have led to erroneous conclusions. For example, the complex and rapidly changing nature of IL-6 mRNA transcription could have led to the conclusion that the microarray results were simply irreproducible, as has been reported for much lower-strength fields [62]. Alternately, the four and five day time points – where IL-6 mRNA levels were actually lower than controls – were not consistent with the strong, multifaceted up-regulation that occurred upon the initial exposure to magnetic stimulus thereby also providing misleading information if regarded in isolation. Therefore, we close by noting the benefits of the robust ability of software tools to uncover signature biological activity – namely, signaling responses associated with IL-6 – even when the key molecular player (i.e., IL-6) is undergoing rapidly changing or oscillatory behavior that would be difficult to understand by itself.

## Conclusion

At the outset, we emphasize that this study was not intended to provide a comprehensive account of the cellular effects of SMF. For example, mechanisms other than those based on a lipid biolayer 'biosensor' may contribute to the transcriptional changes observed in this study as direct effects of SMF on protein-DNA interactions have been postulated [63-66] as have changes in enzymatic and biochemical reactions [5,67-69]. Therefore, we reiterate that our goal was to provide a rudimentary framework of one of many parallel or complementary mechanisms through which magnetic stimuli are transduced from molecular level biosensors into cell-level responses. This objective was pursued by mRNA microarray profiling that verified time-dependent global changes in transcription occurred that were consistent with the activation of signaling pathways. Then, to gain insight into the specific networks affected by SMF exposure, which were not obvious by simple inspection of the genes involved, the microarray data was subject to software analysis and signaling networks consistent with tissue- or organ-level responses to magnetic exposure (that include benefits to wound healing [11] and inflammation [12]; cardiovascular effects [13] such as modulation of blood flow and pressure [10]; and anti-tumor activity [70,71]) were identified.

Although the microarray data identified cellular responses consistent with previously reported biological responses to magnetic exposure, we nonetheless sought to ensure that these associations were not just coincidental or an artefact of the software analysis. Detailed biochemical investigation of all nine pathways (see Table 5) was well beyond the scope of a single study, therefore we selected a single network – IL-6 signaling – for validation and outlined several molecular paths (as shown in Figure 1) that accounted for the multifaceted up-regulation of IL-6 by SMF that occurred over the first 1 to 3 days of exposure. From the standpoint of disease intervention, the up-regulation of IL-6 by SMF at first seems to be unwanted because IL-6 is generally maintained at low levels in healthy tissue [50]. Moreover, chronically elevated levels of IL-6 are usually deleterious (for example, inflammation and unabated astrocyte differentiation associated with increased IL-6 experienced after brain or spinal cord injury blocks axonal regeneration of neurons and thereby hampers full recovery [35]). In some cases, however, the short-term activation of IL-6 can be therapeutically beneficial; for example, this pleiotropic cytokine can be neuroprotective immediately after injury [28]. Consequently, successful therapeutic intervention involving IL-6 is contingent upon transient – as experienced in the SMF-treated cells – rather than on prolonged activation to avoid the deleterious consequences of chronic inflammation and other long term consequences of sustained IL-6 production.

In a final set of experiments, we briefly investigated whether SMF-mediated responses associated with IL-6 signaling translated into changes in phenotype observable at the whole cell level. Although IL-6 impacts numerous cell-level and systemic responses, our experimental efforts were focused by reports that IL-6 guides differentiation of neural stem cells primarily to astrocytes [35]. These clues led us to investigate whether evidence of astrocytogenesis was seen in the hEBD LVEC cells, an embryonic line that is predisposed to neural differentiation [24]. Interestingly, responses consistent with differentiation (i.e., slowed proliferation and morphological changes) were not reflected in the biochemical markers indicative of the astrocyte differentiation expected from IL-6. Instead, markers found in oligodendrocyte precursor cells were manifest, indicating that the other pathways modulated by SMF tuned the 'usual' activity of IL-6. Ultimately, if full oligodendrocyte formation can be promoted *in vivo *by SMF without concomitant astrocyte enhancement (under the current experimental conditions, full differential to oligodendrocytes was not feasible due to the absence of neurons and other glial cells found in the *in situ *oligodendrocyte microenvironment), this capability could lead to non-invasive therapies for conditions such a multiple sclerosis (MS) linked to oligodendrocyte pathologies.

## Methods

### Cell lines and culture conditions

The human embryoid body derived (hEBD) LVEC cell line [24] was obtained from the Shamblott Laboratory (JHMI) and was cultured in EGM2MV media (Clonetics, San Diego, CA) that included 5.0% fetal bovine serum (FBS), hydrocortisone, human basic fibroblast growth factor, human vascular epidermal growth factor, R(3)-insulin-like growth factor I, ascorbic acid, human epidermal growth factor, heparin, gentamycin, and amphotericin. The HEK AD293 line was obtained from the ATCC (Manassas, VA) and incubated in DMEM supplemented with 10% FBS under established conditions [72]. Cells were cultured on tissue culture (T.C.) plastic coated with bovine collagen I (Collaborative Biomedical Products, Bedford, MA; 10 μg/cm^2^) in a water-saturated, 37°C incubator with 5.0% CO_2_.

### Exposure of cultured cells to SMF

Cell exposure to SMF was conducted for time intervals up to a maximum of 9 days using a device obtained from the Advanced Magnetic Research Institute, International (AMRIi, Calgary, AB) that fit into a standard T. C. incubator with sufficient clearance on all sides so that incubator functions (i.e., circulation of CO_2 _and water saturated air) were not affected. The device was designed based on principles derived from clinical testing of SMF (Diabetic Peripheral Neuropathy (ClinicalTrials.gov Identifier: NCT00134524) and Chronic Low Back Pain (NCT00325377)) that the magnetic field must be unidirectional with no reverse field passing through the sample [73]. It was embedded with four 1" × 4" × 6" (inch) permanent neodymium (NdFeB) rectangular block magnets with two located above and two below the central cavity (Additional file 2: Figure S10). The device produced a field with a magnetic flux gradient of < 1.0 mT/mm in the portion of the central cavity where the cells were maintained during experiments. This arrangement contrasts with experimental set-ups where each well of a T. C. plate has been supplied with SMF exposure by using a separate magnet; in these cases (or in experiments specifically designed to test gradient responses) the periphery of the treatment areas were subject to much higher magnetic flux gradients of 20, 21, or 28 mT/mm [31-33,74] that resulted in different cellular responses than observed in the more uniform portions of the magnetic fields.

As just explained, the SMF conditions used in the current experiments were not expected to elicit gradient effects because the gradient used was much shallower than previously reported (i.e., < 1.0 mT/mm compared to 20–28 mT/mm). Nevertheless, to ensure that gradient effects – or other artefacts of the exposure conditions – did not account for the effects observed in hEBD LVEC cells in this study, several control experiments were conducted. First, the direction of the flux (i.e., whether the device was oriented upright so that the field was superimposed on the Earth's magnetic field or oriented upside down so that the applied field countered the background field) was tested and found not to have an impact on the parameters under investigation in hEBD LVEC cells. Second, differences in field strength (i.e., whether the cells were exposed to field at the extreme top or bottom of the treatment device when six T. C. plates – the maximum capacity of the device – were stacked on top of each other) did not measurably affect the outcome of the experimental parameters reported in this study. Finally, the orientation of the T. C. plates (e.g., whether the plates were placed as shown in Figure S10 (Additional file 2) or at 90°, 180°, or 270° angles) did not alter experimental outcomes.

Despite the lack of variation in IL-6 related outcomes, the experiments described in this report were always performed with the induced static magnetic field superimposed in the same direction as the ambient field, the SMF-treated cells were maintained in the central portion of the device oriented as shown in Figure S10 (Additional file 2) in the region the where magnetic flux density ranged between 0.23 and 0.28 T (as measured by a gaussmeter, Type 181002, Thyssen Magnet-und komponententechnik, Dortmund, Germany). Control cells were kept in an identical incubator where the ambient magnetic field was ~52 μT (which was within 1 μT of the background levels measured by us or reported by the National Geophysical Data Center for the location where these experiments were conducted (i.e., 52,359.0 nT at a latitude of 39° 19' 35" and a longitude of – 76° 36' 17",. http://ngdc.noaa.gov/geomagmodels/IGRFWMM.jsp?defaultModel=IGRF).

### Transcriptional (mRNA) profiling

In all cases, the cells subjected to microarray profiling were obtained from the same initial culture batch and were subsequently cultured for a total of six days before RNA was isolated and analyzed. Subconfluent (70%–80%) undifferentiated hEBD LVEC (passage 11) cells were trypsinized, resuspended, and replated at 5.0 × 10^5 ^cells in 10 ml medium in 10 cm T. C. dishes on "Day 0". All cells were allowed to recover from the plating process by incubating them under normal culture conditions for one day after which time four conditions were investigated. For **Group 1**, control cells were incubated for five additional days under normal culture conditions. For **Group 2**, cells were cultured under normal conditions for four additional days and then subjected to one day (~24 h) of SMF exposure in the magnetic treatment device on "Day 6"; mRNA from these cells, as well as from the third group, was isolated for analysis immediately after magnetic exposure ended. For **Group 3**, cells were subjected to continuous SMF exposure for five days. Finally, for **Group 4**, cells were exposed to SMF for four days (from Day 2 through Day 5) followed by a 24 h recovery period during which time they were incubated under normal culture conditions. mRNA was isolated from the cells and microarray analysis was done using the Affymetrix Human Genome U133 2.0 Plus Chip using the protocols and facilities available through the Johns Hopkins Cancer Center Microarray Core. All data was obtained in duplicate from independent experiments. Software analysis was performed using the Ingenuity Pathway tool (available through the Microarray Core) using merged data from each set of independent experiments. The microarry data have been deposited in NCBI's Gene Expression Omnibus [75] and are accessible through GEO Series accession number GSE14474 http://www.ncbi.nlm.nih.gov/geo/query/acc.cgi?acc=GSE14474.

### Calcium measurements

hEBD LVEC cells were incubated in Ca^2+^- and Mg^2+^-free for PBS for up to four hours (longer time points decreased cell viability making the assay results unreliable) with or without SMF exposure. Intra- and extra-cellular calcium levels were measured separately after the cells were separated from their supernatants by pelleting with a 300 g, 2 min centrifugation step followed by lysis by sonication with a GE130PB ultrasonic processor (General Electric, New York, NY). Analysis of the supernatants and cell lysates was then conducted using a calcium reagent set (Pointe Scientific Inc., Canton, MI) and published methods [76].

### Treatment of cells with exogenous gangliosides (GM3 and GD3)

The basic procedure for ganglioside supplementation followed published procedures [77]. Briefly, cells were plated in 6-well tissue culture dishes and incubated until they reached 60% confluence. GM3 or GD3 (Matreya LLC, Pleasant Gap, PA) was resuspended in serum-free medium and briefly sonicated to ensure appropriate micellar suspension and cellular incorporation of these gangliosides. Cells were then incubated in culture medium containing 1.0, 5.0, 10, 20, or 50 μM GM3 or GD3 for varying periods of times (as specified in the Results section and accompanying figures). In each case, results were compared with a "solvent control," where an equal volume of medium was added to cells without ganglioside.

### Real-time PCR quantification of gene expression

The mRNA *levelsofST*3*GAL*5, *NEU*3, *TLR*4, *IL*-6*andglyceraldehyde*-3-*phosphatedehydrogenase*(*GAPDH*)*wereanalyzedbyquantitativereal*-*timepolymerasechain *reaction (qRT-PCR) [77,78]. Primers (listed in Table 6), were designed by using the Primer3 software made available through the Broad Institute http://genecruiser.broadinstitute.org/science/software and obtained from MWG-Biotech (High Point, NC). The basic protocol followed for qRT-PCR experiments began with the isolation of total RNA from 5.0 × 10^6 ^cells with the RNeasy Mini Kit (Qiagen, Valencia, CA) or by the TRIzol (Invitrogen) method. RNA quality was assessed by agarose gel electrophoresis (1.8% gels run with TAE buffer followed by nucleic acid band visualization under UV illumination after ethidium bromide staining) and quantified by A_260_/A_280 _OD readings. RNA integrity was confirmed using 18 S rRNA primers, and samples were standardized based on equal levels of β-actin cDNA. Quantitative real-time PCR was performed in an ABI Prism 7000 sequence detector (Applied Biosystems) using SYBR Green PCR Master Mix reagent (Applied Biosystems). Reactions were performed in 20 μl of a mixture containing a 2.0 μl cDNA dilution, 1.0 μl (10 pmol/μl) of primer, and 10 μl of 2× SYBR master mix containing Amplitaq Gold DNA polymerase, reaction buffer, a dNTP mixture with dUTP, passive reference, and the SYBR Green I. qRT-PCR conditions were as follows: one cycle of 2.0 min at 50°C, 95°C for 10 min, followed by 40 cycles of 95°C for 15 s and 60°C for 1.0 min. Specific PCR products were detected with the fluorescent double-stranded DNA binding dye, SYBR Green. qRT-PCR amplification was performed in quadruplicate for each sample (typically values for the replicates were within 2% of each other) and the results were replicated in at least three independent experiments. Gel electrophoresis and melting curve analyses were performed to confirm correct PCR product sizes and the absence of nonspecific bands. The expression level of each gene was normalized against β-actin using the comparative CT method [79] according to the manufacturer's protocols.

**Table 6:** Primers used for qRT-PCR

Gene	Forward primer (5' to 3')	Reverse primer (5' to 3')
ST3GAL5	CCC TGA ACC AGT TCG ATG TT	CAT TGC TTG AAG CCA GTT GA
NEU3	CCT GAA GCC ACT GAT GGA A	TTC CTG CCT GAC ACA ATC TG
IL-6	TAC ATC CTC GAC GGC ATC TC	GCT ACA TTT GCC GAA GAG CC
TLR4	TGA GCA GTC GTG CTG GTA TC	CAG GGC TTT TCT GAG TCG T
GAPDH	GCA AAT TCC ATG GCA CCG T	TCG CCC CAC TTG ATT TTG G

### Analysis of cell surface and total levels of GM3 and GD3

The method used for the analysis of cell surface GM3 and GD3 expression by flow cytometry was adapted from published protocols [77]. Briefly, these tests were performed by detaching hEBD LVEC cells by trypsinization and washing them with washing buffer (1.0% bovine serum albumin, 0.1% NaN_3 _in phosphate-buffered saline). Cells (1.0 × 10^6^) were stained with 20 μg/ml of a mouse monoclonal antibody against GM3 (NBT-M101/M102, isotype IgM, clone M2590; Cosmo Bio Co., Ltd., Tokyo, Japan) and detected with fluorescein isothiocyanate-conjugated Affinipure rabbit anti-mouse IgM (Jackson Immunoresearch, West Grove, PA). A similar procedure was used for GD3 analysis, except cells were stained with mouse anti-human ganglioside GD3 monoclonal antibody (Product number 371440, clone 110.14F9, isotype IgG3; Calbiochem) diluted 1:50 in washing buffer and detected with a donkey anti-mouse IgG antibody conjugated to fluorescein (Jackson Immunoresearch). Control samples stained with secondary antibody alone were analyzed in parallel in each experiment. Samples were analyzed with a FACScan flow cytometer and Cell Quest software (BD Immunocytometry Systems, San Diego, CA), and a minimum of 5000 events were acquired for each sample. Analysis of total (i.e., surface and intracellular) GM3 and GD3 was tested in fixed and permeabilized cells [80] by adaptation of a method used to quantify intracellular levels of p21^WAF1 ^[81]. Briefly, before completing the staining procedure described above, cells were fixed by incubation in 4.0% paraformaldehyde in phosphate-buffered saline for 10 min at room temperature followed by

### Western blot analyses

An equal amount of protein from each sample (20 μg) was incubated for 5.0 min at 100°C in Laemmli buffer (Bio-Rad), separated on a 7–11% SDS-polyacrylamide discontinuous gel, and then electrophoretically transferred to a nitrocellulose membrane (Bio-Rad). The membrane was blocked with Tris-buffered saline containing 5.0% nonfat milk and 0.1% Tween 20 for 1.0 h at room temperature and then incubated overnight with rabbit phospho-p44/42 MAPK (i.e., pERK1/2) monoclonal antibody and p44/42 MAPK (i.e., ERK1/2) antibody (1:1000 dilution) or phospho-p38 MAPK and p38 MAPK (1:2000) or phospho-stat3 (Tyr705) and Stat3 rabbit antibody (1:1000) (Cell Signaling Technology, Beverly, MA) at 4.0°C, followed by anti-rabbit or anti-mouse IgG, horseradish peroxidase-linked antibody (1:2000) for 1.0 h. Bound antibody on the membrane was detected using the SuperSignal West Dura Extended Duration Substrate (Pierce) according to the protocols supplied by the manufacturer. Quantification of bands was performed by using the NIH ImageJ software (available on the World Wide Web at rsb.info.nih.gov/nih-image) following a published method [82,83].

### Measurement of proliferation with the MTT assay

For proliferation assays, control or SMF-exposed hEBD LVEC cells were added to 96-well tissue culture plates at 3000 cells/well in serum-containing medium and cultured for up to nine days with the culture medium replenished every 3^rd ^day. To quantify cell proliferation by measuring metabolic activity, 3-(4,5-Dimethylthiazol-2-yl)-2,5-diphenyltetrazolium bromide (MTT, (Sigma) was added to each well (0.5 mg/ml). After incubation for 3.0 h at 37°C, the supernatants were aspirated, and 100 μl of *n*-propyl alcohol containing 0.1% Nonidet P-40 and 4.0 mM HCl were added. The colorimetric reaction was quantified by using an automatic plate reader, μ Quant (Bio-tek Instrument Inc., Winooski, VT) to measure absorbance at 570 nm with a reference filter of 690 nm. Each MTT assay was carried out in triplicate. In all cases, measurement of proliferation through cell counting by using a Coulter Z2 instrument (as described in our previous publications [84]) yielded identical results.

### Detection of apoptosis by Annexin V/propidium iodide assays

The Annexin V/propidium iodide flow cytometry method was used for the detection and quantification of apoptosis by following the procedure previously reported for Jurkat cells [85,86] with the added step of trypsinizing the adherent hEBD LVEC cells (the previously-tested Jurkat cells grow in suspension and did not require this step). After trypsinization and resuspension in complete medium, cells were counted with a Coulter Z2 instrument, 1.0 × 10^6 ^cells from each sample were pelleted by centrifugation, washed by gentle resuspension in Dulbecco's phosphate-buffered saline, centrifuged again, and suspended in staining buffer. The cells were stained with fluorescein isothiocyanate-labeled Annexin V and propidium iodide and analyzed by flow cytometry as described previously [85,86].

### Measurement of secreted IL-6 by ELISA

Cells were seeded in triplicate in a 96 well culture plate at 6000 cells/well in 200 μl of medium. After two days, cells were exposed to SMF and supernatant was collected over the time course indicated in the Results section and the concentration of IL-6 was determined by an ELISA kit designed for this purpose (eBioscience, San Diego, CA) following the protocol provided by the manufacturer.

### Confocal imaging of Gal-C, GFAP, NEF and Vim

hEBD LVEC cells (1.0 × 10^5 ^in 2.0 ml of medium) were plated on collagen coated 35 mm glass bottom dishes (35 mm, MatTek Corporation, Ashland, MA) and either exposed to SMF during culture or subject to normal cultivation. On day 4 the monolayers were fixed in reagent A and permeabilized in reagent B (Fix & Perm, Reagents A and B, Caltag Laboratories, Burlingame, CA) followed by washing with 3.0% BSA in PBS. Cells were incubated with anti-galactocerebroside (Gal-C, 1:100) (Sigma, Saint Louis, MO); anti-NEF 70 kD (1:100) (Chemicon, Temecula, CA); anti-GFAP (1:100) (Santa Cruz, Los Angeles, CA); or anti-vimentin (anti-Vim, 1:500) (BD Pharmingen, San Diego, CA) for 2.0 h at RT. The secondary antibody used to stain anti-GFAP, anti-NEF, and anti-Vim was Cy3-conjugated Donkey anti-mouse IgG(H+L) and Cy3–conjugated Donkey anti-Rabbit IgG(H+L) was the secondary anti-body used for anti-Gal-C (both were obtained from Jackson ImmunoResearch Labs, West Grove, PA and used at a 1:100). In all cases, a solution of the high-affinity probe for F-actin Oregon Green^® ^488 phalloidin (1:100) (Molecular Probes, now Invitrogen, Eugene, OR, Cat. No. O7466) was added to the monolayers and incubated for 20 min and the monolayers were stained with nuclear-localizing dye DAPI (1.0 μg/mL) for 10 min at RT. The monolayers were then mounted using ProLong Gold^® ^anti-fade reagent (Molecular Probes, Eugene, OR, Cat. No.P36934) and imaged by using a Zeiss 510 Meta confocal microscope.

## List of abbreviations

**GD3**: ganglioside GM3 (Neu5Acα3Galβ4GlcCer); **GM3**: ganglioside GD3 (Neu5Acα8Neu5Acα3Galβ4GlcCer); **hEBD LVEC**: the human embryoid body derived LVEC cell line; **IL-6**: interleukin-6; **LacCer**: lactosylceramide (Galβ4GlcCer); **NEU3**: neuramindase 3; **qRT-PCR**: quantitative real-time polymerase chain reaction; **SD**: standard deviation; **SMF**: static magnetic field(s); **ST3GAL5**: β-galactoside α-2,3-sialyltransferase 5; **TLR4**: toll-like receptor 4.

## Authors' contributions

KJY was the principal investigator on this project, AS performed the microarray experiments and analysis, ZW designed, supervised, and carried out the majority of the cell, molecular, and biochemical assays performed in this study (P-LC provided substantial assistance in the design and execution of these experiments).

## Supplementary Material

Additional file 1 **Annotation of the signaling networks identified to respond to SMF exposure in hEBD LVEC cells**. The Ingenuity Pathway Analysis software tool was used to annotate the networks listed in Table 5 and the resulting diagrams are provided in Figures S1 through S9 (corresponding to ID#1–9, respectively). Click here for file

Additional file 2 **Description of the SMF treatment device**. The device used to treat cells with 0.23 to 0.28 T static magnetic fields is shown (in Figure S10) along with field orientation and strength. Click here for file
