# Supplementary material for: Moderate strength (0.23–0.28 T) static magnetic fields (SMF) modulate signaling and differentiation in human embryonic cells
Source: BMC Genomics. 2009 Aug 4;10:356. doi: 10.1186/1471-2164-10-356 (PMC2907690; doi:10.1186/1471-2164-10-356)
Supplement: Additional file 1 — Annotation of the signaling networks identified to respond to SMF exposure in hEBD LVEC cells. The Ingenuity Pathway Analysis software tool was used to annotate the networks listed in Table 5 and the resulting diagrams are provided in Figures S1 through S9 (corresponding to ID#1–9, respectively). [file 1471-2164-10-356-S1.ppt]

## Slide 1
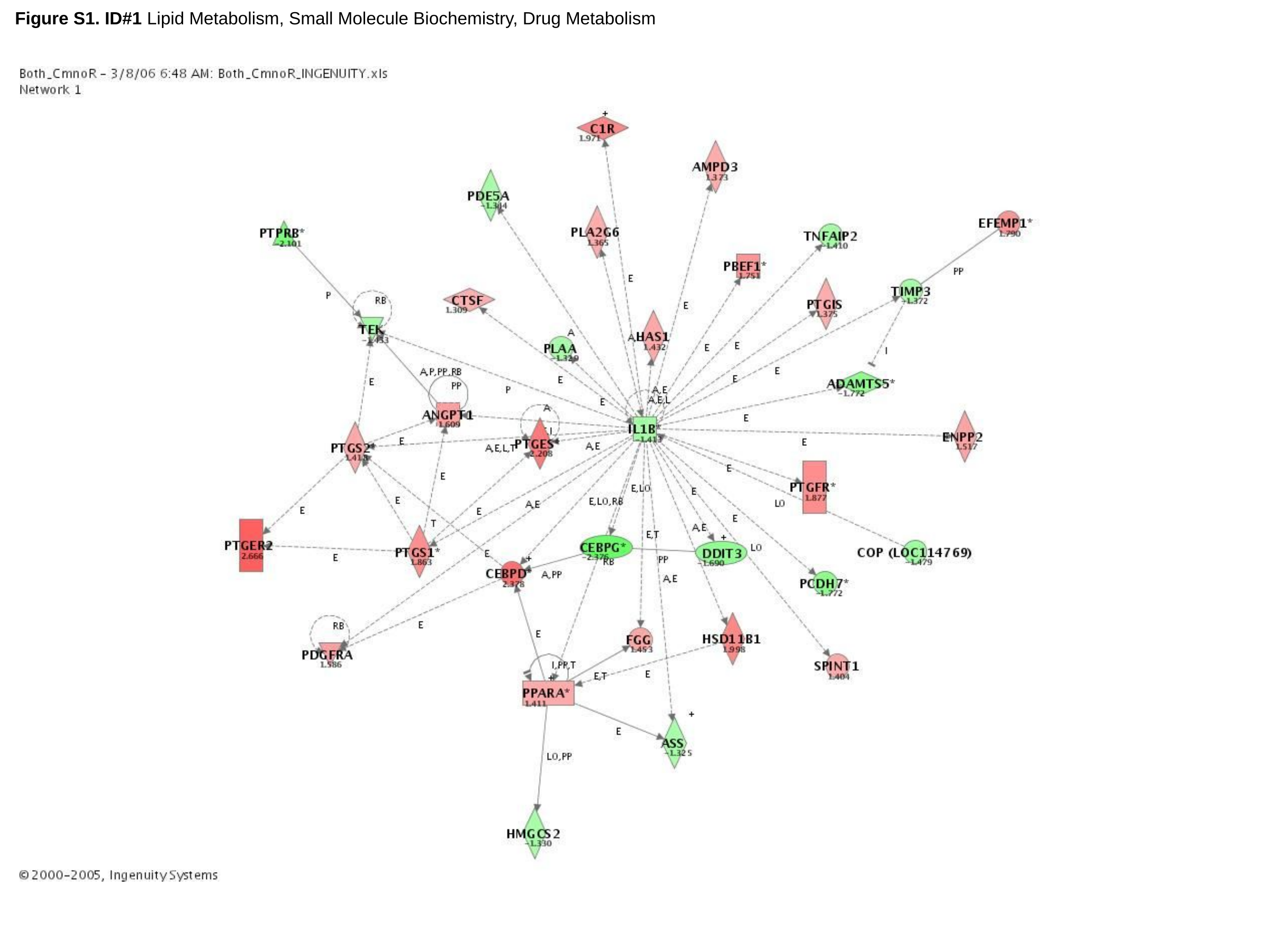

Figure S1. ID#1 Lipid Metabolism, Small Molecule Biochemistry, Drug Metabolism

## Slide 2
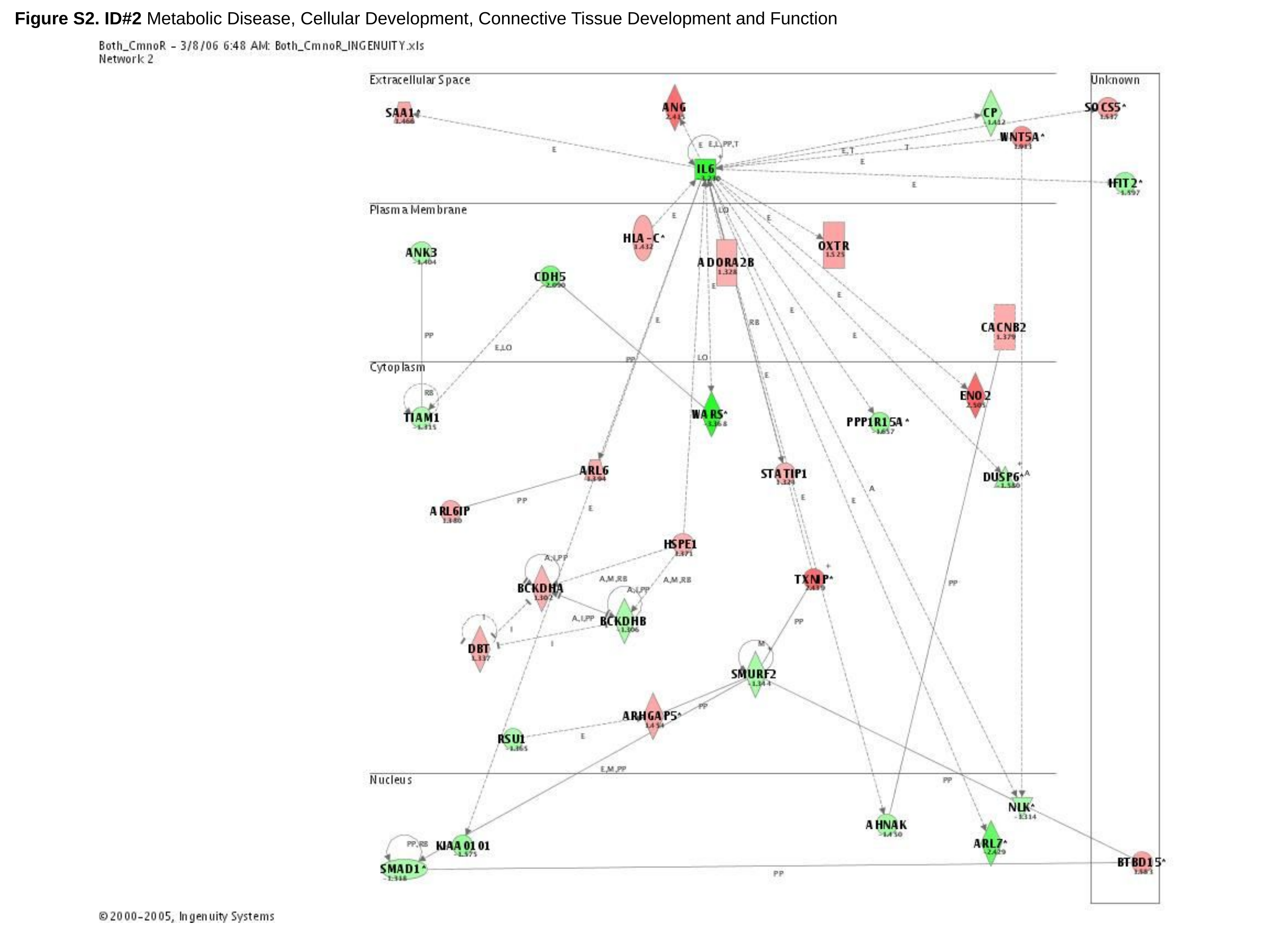

Figure S2. ID#2 Metabolic Disease, Cellular Development, Connective Tissue Development and Function

## Slide 3
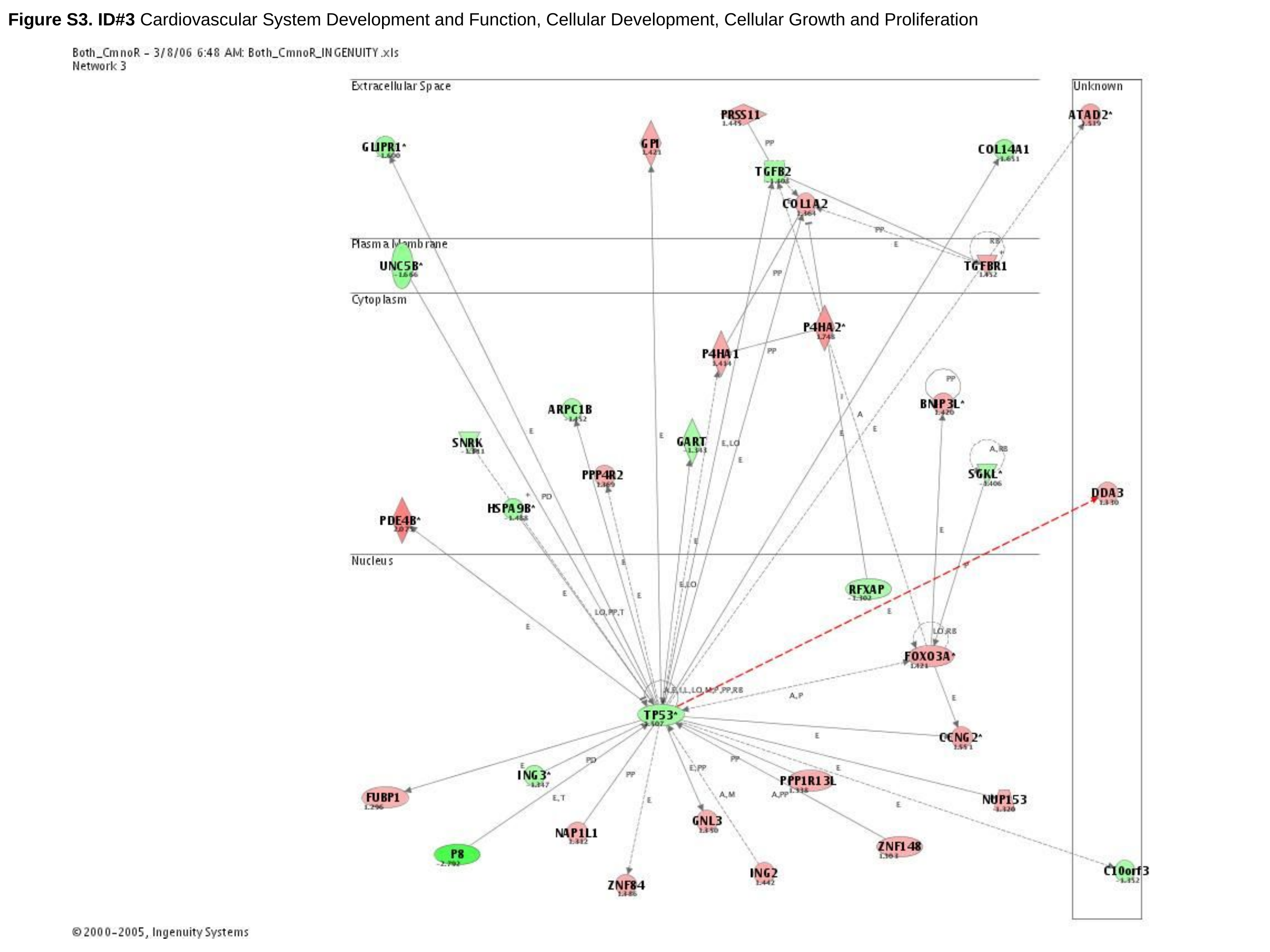

Figure S3. ID#3 Cardiovascular System Development and Function, Cellular Development, Cellular Growth and Proliferation

## Slide 4
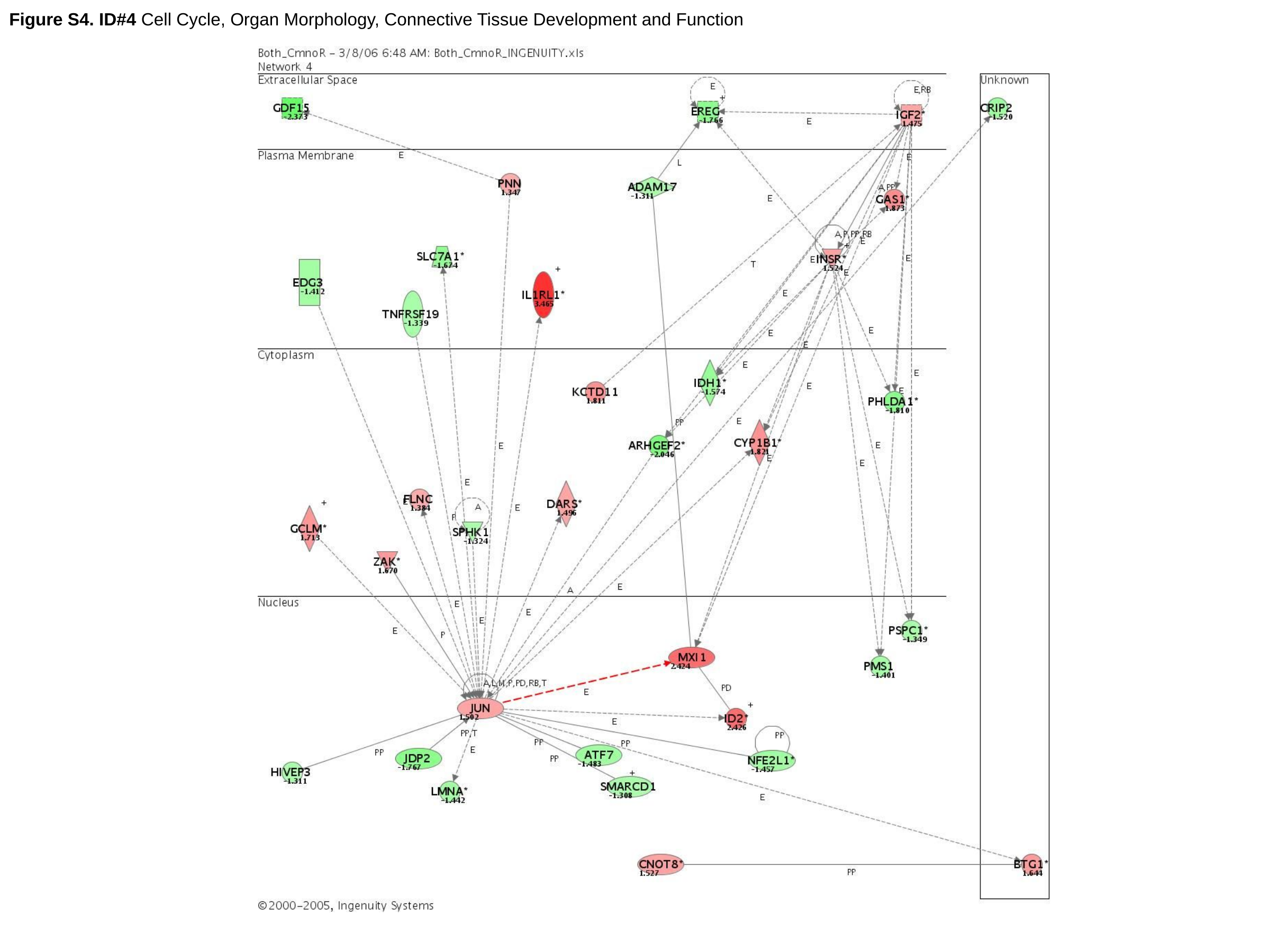

Figure S4. ID#4 Cell Cycle, Organ Morphology, Connective Tissue Development and Function

## Slide 5
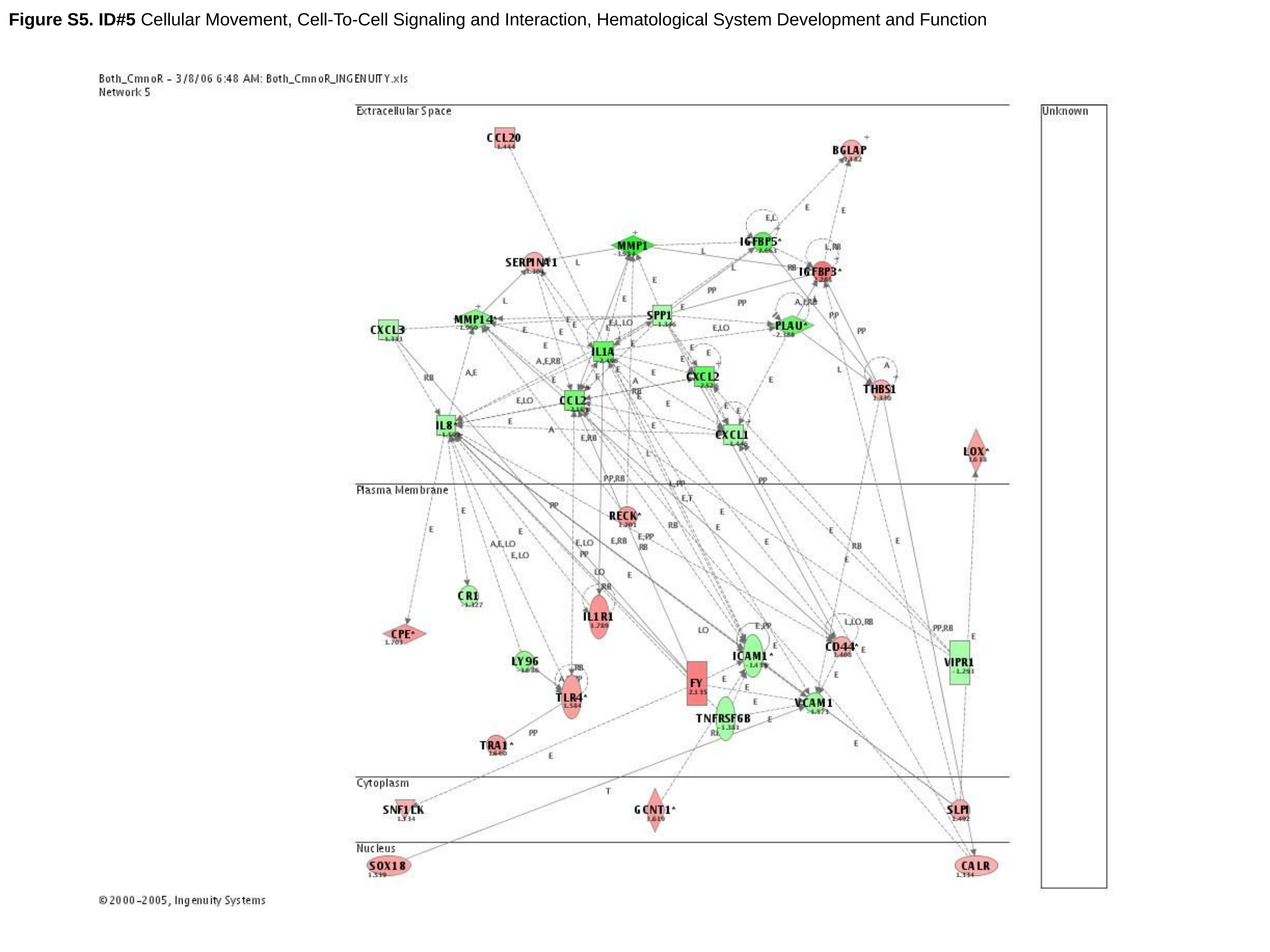

Figure S5. ID#5 Cellular Movement, Cell-To-Cell Signaling and Interaction, Hematological System Development and Function

## Slide 6
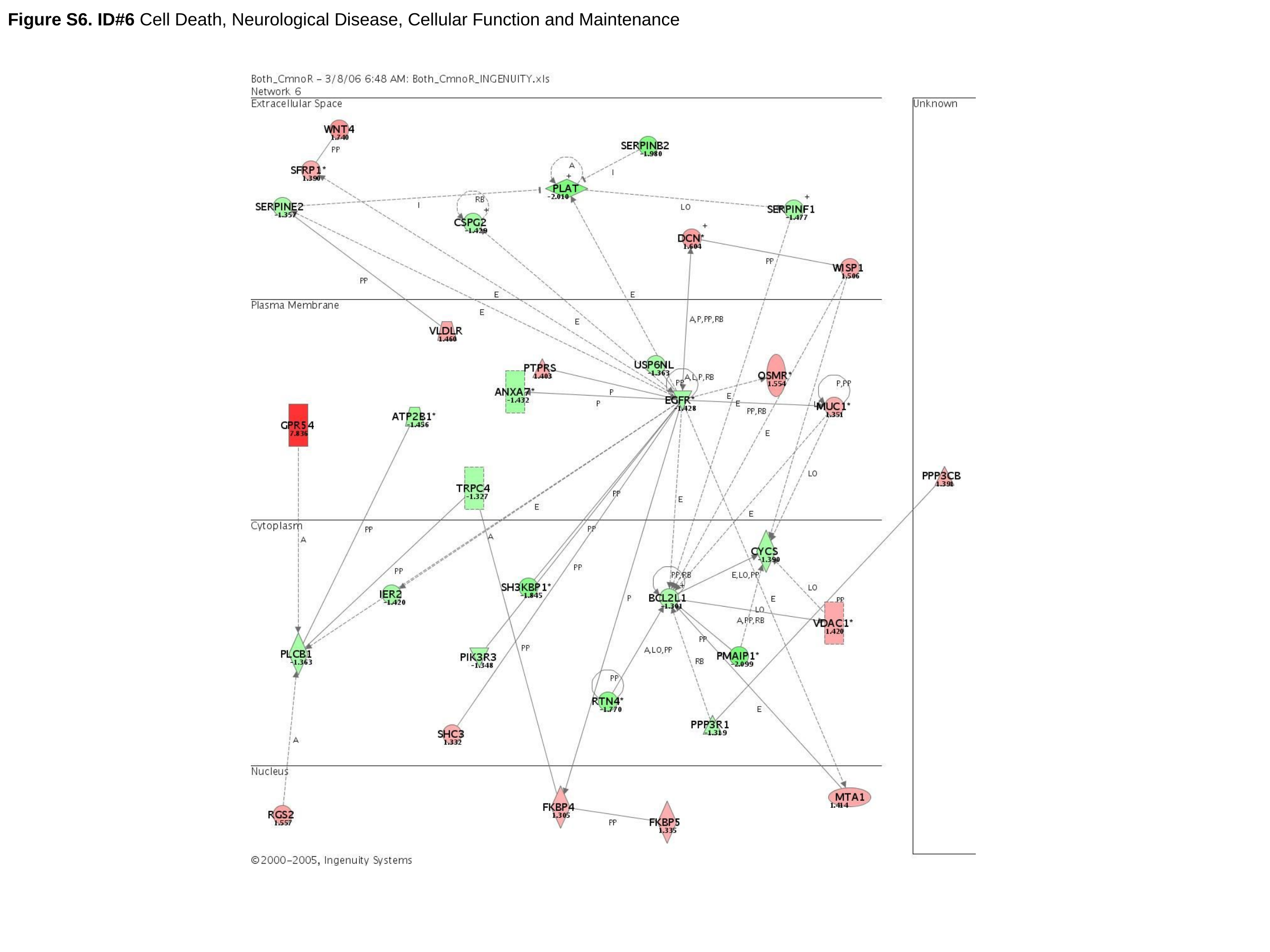

Figure S6. ID#6 Cell Death, Neurological Disease, Cellular Function and Maintenance

## Slide 7
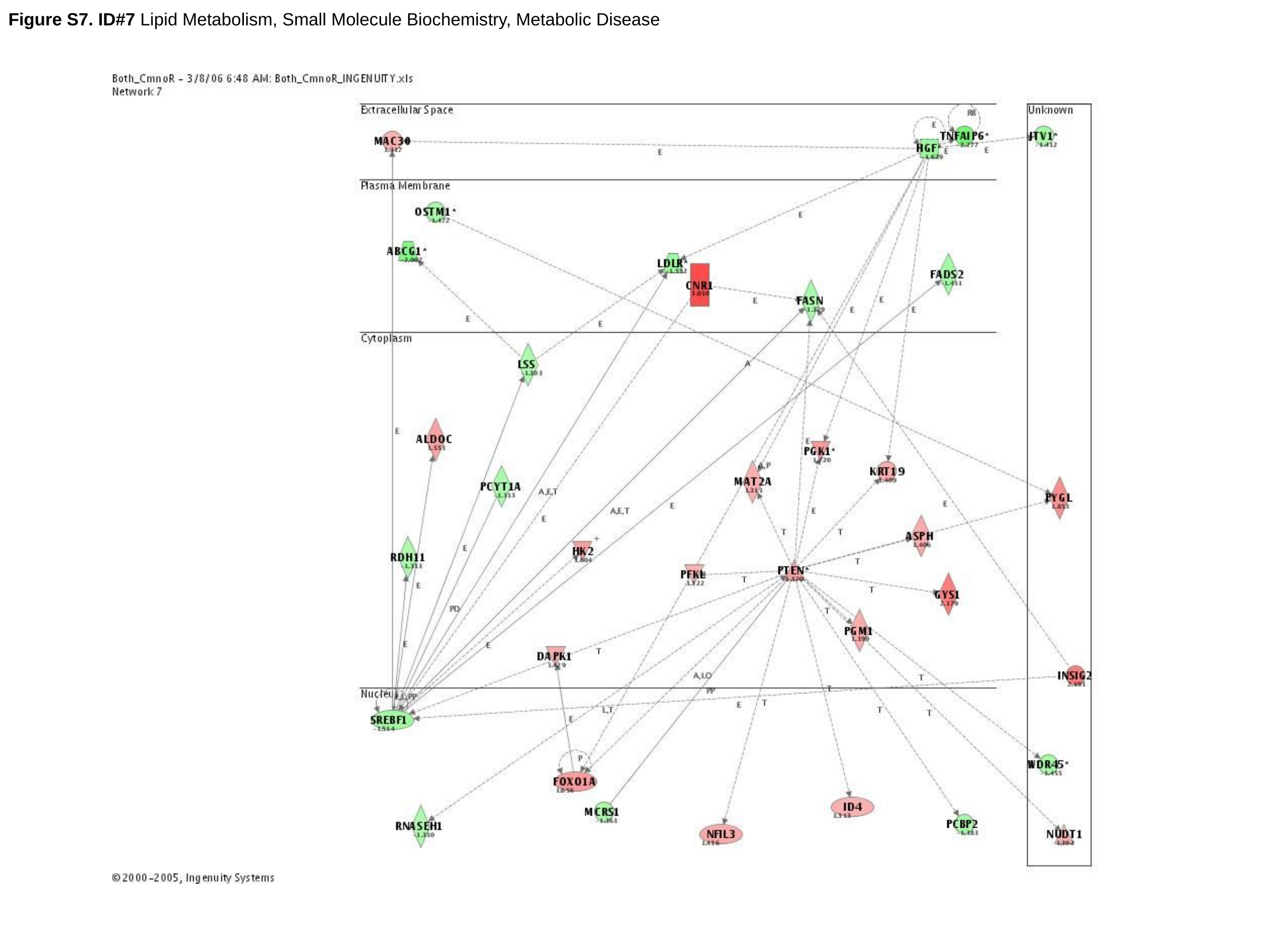

Figure S7. ID#7 Lipid Metabolism, Small Molecule Biochemistry, Metabolic Disease

## Slide 8
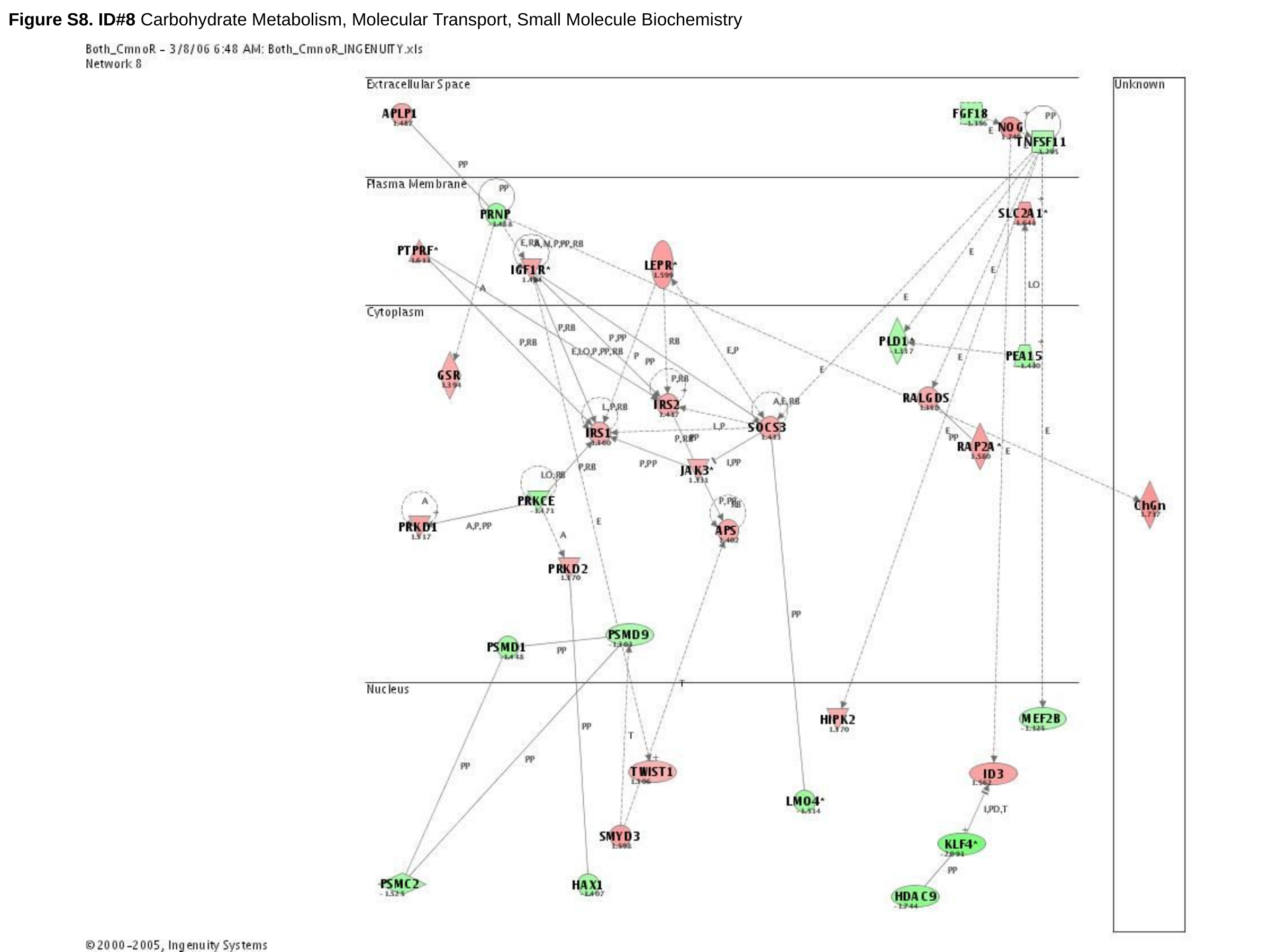

Figure S8. ID#8 Carbohydrate Metabolism, Molecular Transport, Small Molecule Biochemistry

## Slide 9
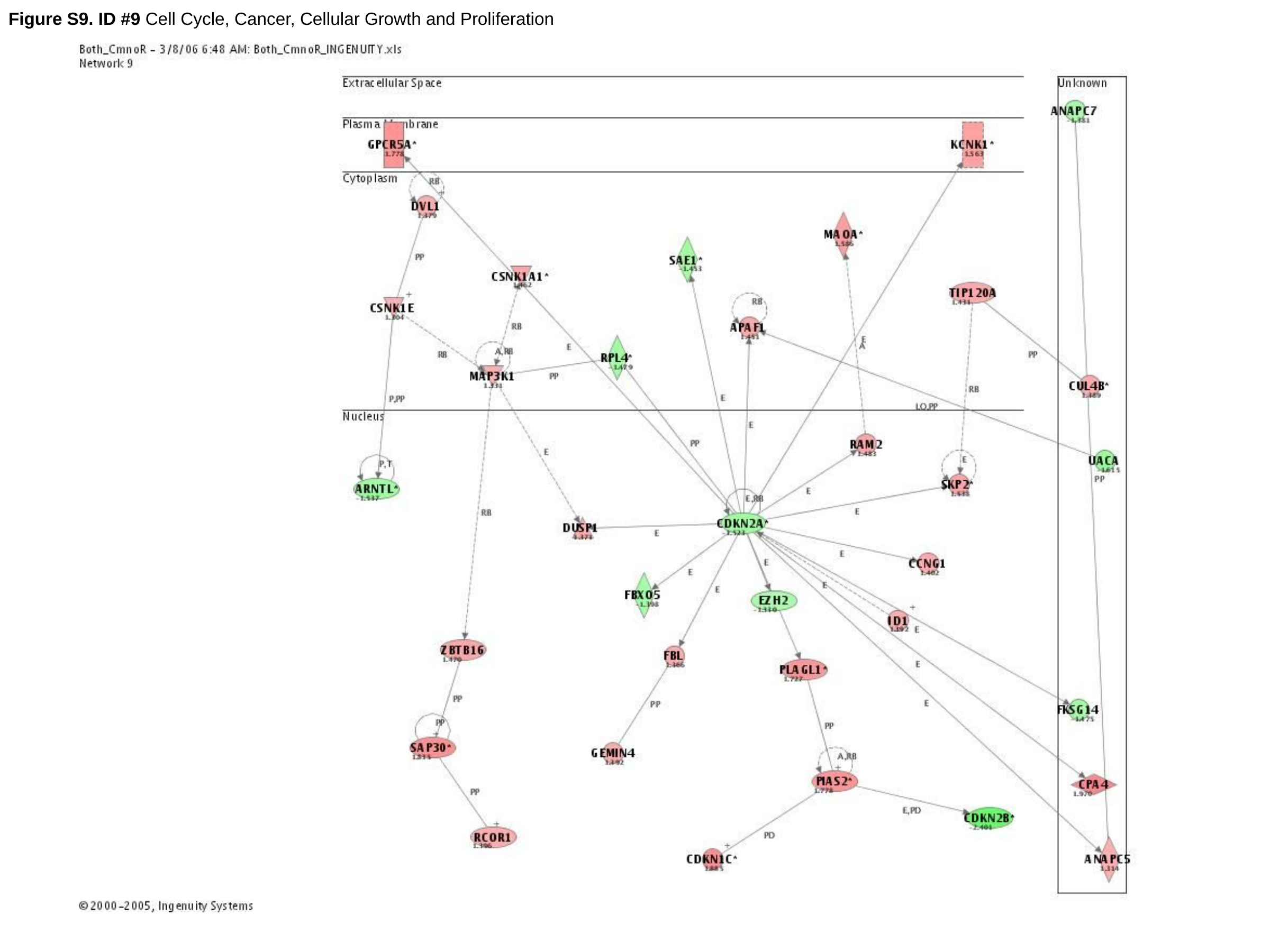

Figure S9. ID #9 Cell Cycle, Cancer, Cellular Growth and Proliferation
