# Supplementary material for: Moderate strength (0.23–0.28 T) static magnetic fields (SMF) modulate signaling and differentiation in human embryonic cells
Source: BMC Genomics. 2009 Aug 4;10:356. doi: 10.1186/1471-2164-10-356 (PMC2907690; doi:10.1186/1471-2164-10-356)
Supplement: Additional file 2 — Description of the SMF treatment device. The device used to treat cells with 0.23 to 0.28 T static magnetic fields is shown (in Figure S10) along with field orientation and strength. [file 1471-2164-10-356-S2.ppt]

## Slide 1
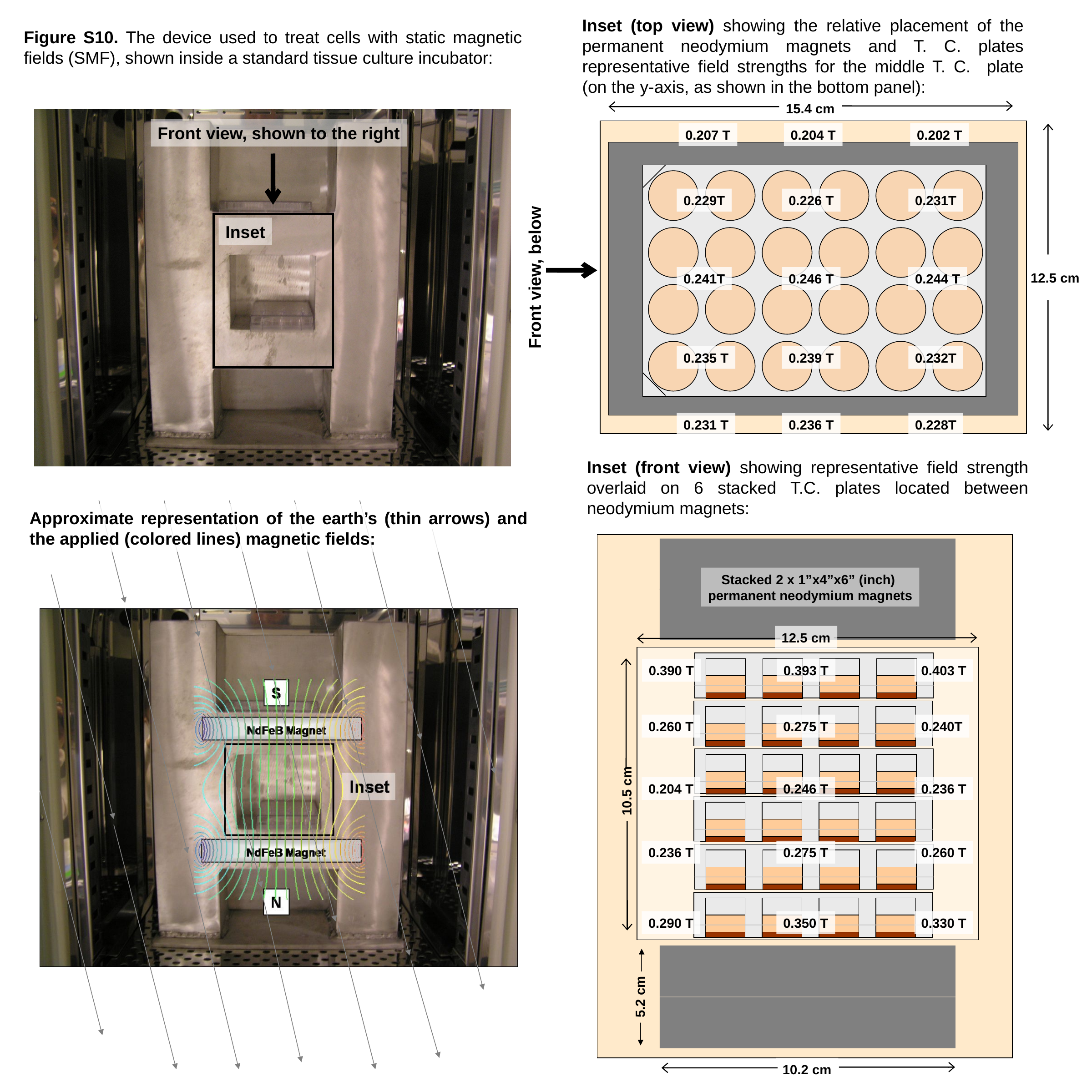

Inset (top view) showing the relative placement of the permanent neodymium magnets and T. C. plates representative field strengths for the middle T. C. plate (on the y-axis, as shown in the bottom panel):
Figure S10. The device used to treat cells with static magnetic fields (SMF), shown inside a standard tissue culture incubator:
15.4 cm
Inset
Top view
Front view, shown to the right
0.207 T
0.204 T
0.202 T
0.229T
0.226 T
0.231T
Front view, below
12.5 cm
0.241T
0.246 T
0.244 T
0.235 T
0.239 T
0.232T
0.231 T
0.236 T
0.228T
Inset (front view) showing representative field strength overlaid on 6 stacked T.C. plates located between neodymium magnets:
Approximate representation of the earth’s (thin arrows) and the applied (colored lines) magnetic fields:
Stacked 2 x 1”x4”x6” (inch)
permanent neodymium magnets
12.5 cm
0.390 T
0.393 T
0.403 T
0.260 T
0.275 T
0.240T
0.204 T
0.246 T
0.236 T
10.5 cm
0.236 T
0.275 T
0.260 T
0.290 T
0.350 T
0.330 T
5.2 cm
10.2 cm
